# Hydrostatic pressure prevents chondrocyte differentiation through heterochromatin remodeling

**DOI:** 10.1242/jcs.247643

**Published:** 2021-01-27

**Authors:** Koichiro Maki, Michele M. Nava, Clémentine Villeneuve, Minki Chang, Katsuko S. Furukawa, Takashi Ushida, Sara A. Wickström

**Affiliations:** 1 Helsinki Institute of Life Science, Biomedicum, University of Helsinki, 00290 Helsinki, Finland; 2 Wihuri Research Institute, Biomedicum, University of Helsinki, 00290 Helsinki, Finland; 3 Stem Cells and Metabolism Research Program, Faculty of Medicine, University of Helsinki, 00290 Helsinki, Finland; 4 Department of Mechanical Engineering, The University of Tokyo, Tokyo 113-0033, Japan; 5 Max Planck Institute for Biology of Ageing, 50931 Cologne, Germany; 6 Cologne Excellence Cluster for Stress Responses in Ageing-associated diseases (CECAD), University of Cologne, 50931 Cologne, Germany

**Keywords:** Chondrocyte, Differentiation, Heterochromatin, Hydrostatic pressure, Mechanotransduction, Nucleus, Replicative stress

## Abstract

Articular cartilage protects and lubricates joints for smooth motion and transmission of loads. Owing to its high water content, chondrocytes within the cartilage are exposed to high levels of hydrostatic pressure, which has been shown to promote chondrocyte identity through unknown mechanisms. Here, we investigate the effects of hydrostatic pressure on chondrocyte state and behavior, and discover that application of hydrostatic pressure promotes chondrocyte quiescence and prevents maturation towards the hypertrophic state. Mechanistically, hydrostatic pressure reduces the amount of trimethylated H3K9 (K3K9me3)-marked constitutive heterochromatin and concomitantly increases H3K27me3-marked facultative heterochromatin. Reduced levels of H3K9me3 attenuates expression of pre-hypertrophic genes, replication and transcription, thereby reducing replicative stress. Conversely, promoting replicative stress by inhibition of topoisomerase II decreases Sox9 expression, suggesting that it enhances chondrocyte maturation. Our results reveal how hydrostatic pressure triggers chromatin remodeling to impact cell fate and function.

This article has an associated First Person interview with the first author of the paper.

## Abstract (precis)

**Highlighted Article:** Hydrostatic pressure promotes chondrocyte quiescence and immature chondrocyte state through reducing the amount of H3K9me3-marked heterochromatin.

## INTRODUCTION

The ends of long bones at the regions of joints are covered by a specialized connective tissue termed articular cartilage; a resilient, smooth and elastic tissue with a low frictional coefficient that protects and lubricates joints for smooth motion and transmission of loads. Articular cartilage lacks blood vessels, lymphatics and nerves, and despite being subject to a harsh biomechanical environment, it has limited capacity for intrinsic healing and repair. In this regard, the preservation of healthy articular cartilage is of critical importance for joint function (Kobayashi and Kronenberg, 2014). Cartilage is formed through a process termed chondrogenesis, which proceeds through mesenchymal cell condensation and subsequent chondroprogenitor cell differentiation. Following chondrogenesis, the chondrocytes remain as resting, quiescent cells forming the cartilage tissue. Alternatively, chondrocytes undergo proliferation and terminal differentiation to hypertrophic chondrocytes, and subsequently osteoblasts, to form bone in a process termed endochondral ossification (Tsang and Cheah, 2019).

In the late embryo and at early neonatal stages of mouse development, the articular cartilage is a compact, highly cellular tissue with isotropic distribution of cells. During subsequent postnatal development and growth, the articular cartilage grows in thickness, the chondrocytes enlarge in size, extracellular matrix becomes abundant and the tissue eventually acquires its zonal anisotropic organization (Kozhemyakina et al., 2015; Tsang and Cheah, 2019). The surface zone of articular cartilage is made of flat cells oriented along the main axis of movement and producing lubricating molecules. This surface zone contains the cartilage progenitor cells (Dowthwaite et al., 2004). Below the surface zone, the medial and deep zone contain large round chondrocytes surrounded by typical cartilage matrix that are arranged in vertical rows (Kozhemyakina et al., 2015). How these sequential differentiation and cellular reorganization steps are regulated is unclear.

Cartilage is comprised of 70–80% water per wet mass, making it a highly hydrated tissue. The high water content is maintained by abundant proteoglycans present in the matrix. During joint loading, uniform normal stress is exerted on chondrocytes. As water is trapped within the tissue matrix, pressurization of the fluid initially supports the applied load. Eventually, fluid is expelled from the tissue, and the frictional force between the fluid and solid phases of the tissue dissipates energy from the applied load. In the joint, cartilage is typically exposed to stresses between 0.2 and 10 MPa (Schinagl et al., 1997; Urban, 1994). These stresses are translated to hydrostatic pressure (HP) due to fluid phase pressurization, as described above. Mechanical loading of cartilage has been recognized as an important contributor to chondrocyte identity, and, for example, bioreactors that mechanically load the tissue improve the efficiency of chondrocyte differentiation from mesenchymal stem cells (Elder and Athanasiou, 2009). However, the precise mechanisms by which HP enhances chondrogenesis are unclear.

Here, we investigate the effects of physiological levels of HP on chondrocyte state and behavior. We discover that application of cyclic hydrostatic pressure promotes chondrocyte quiescence and a Sox9-high immature state through a reduction of trimethylated H3K9 (H3K9me3)-marked constitutive heterochromatin, with a concomitant increase in H3K27me3-marked facultative heterochromatin. This quiescent, Sox9-high state counteracts replicative stress and DNA damage. Conversely, promoting replicative stress by inhibition of topoisomerase II decreases Sox9 levels, suggesting DNA damage might enhance chondrocyte maturation. Our results reveal how hydrostatic pressure triggers chromatin remodeling to impact cell fate and function.

## RESULTS

### Hydrostatic pressure promotes an immature quiescent state of chondrocytes

**Fig. 1.**
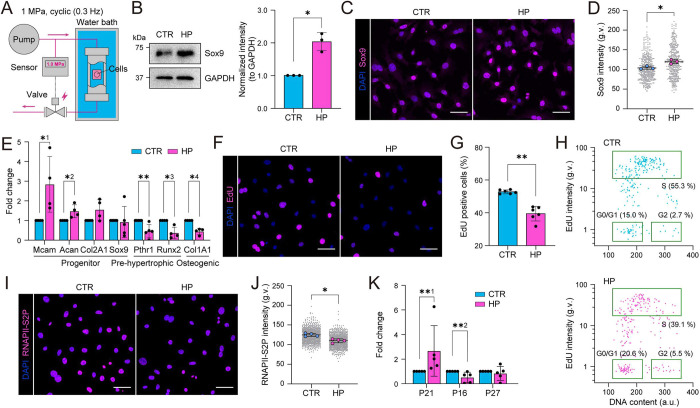
**Hydrostatic pressure promotes a quiescent progenitor state of chondrocytes.** (A) Schematic illustration of the experimental design for cyclic hydrostatic pressure (HP) application where a liquid chromatography pump is combined with a solenoid valve and a pressure sensor to induce cyclic HP with a 1 MPa threshold on cells in a water bath. (B) Representative western blots and quantification for Sox9 from cells exposed to cyclic HP for 6 h (*n*=3 independent experiments). *P=0.0217 (one sample *t*-test). (C) Representative Sox9 (magenta) and nuclear (DAPI; blue) immunofluorescence images of cells exposed to cyclic HP for 6 h. (D) Quantification of immunofluorescence images in C showing an increase in Sox9 intensity in response to HP (*n*=3 independent experiments with >120 cells/condition/experiment). *P=0.0217 (Mann–Whitney test). (E) qPCR analysis for chondrocyte progenitor markers Mcam, Acan, Col2A1 and Sox9, and pre-hypertrophic and osteogenic differentiation markers Pthr1, Runx2, Col1A1 in HP loaded and corresponding control samples (*n*=4 or 5 independent experiments). *^1^*P*=0.0286; *^2^*P*=0.0286; *^3^*P*=0.0286; *^4^*P*=0.0286; **P=0.0079 (Mann–Whitney test). (F) Representative EdU (magenta) and nuclear (DAPI; blue) images of cells exposed to cyclic HP for 6 h. (G) Quantification of images in F showing a decrease in EdU-positive cells (%) in response to HP (*n*=6 independent experiments with >255 cells/condition/experiment). **P=0.0022 (Mann–Whitney test). (H) Scatter plots of EdU intensity versus DNA content (from DAPI stain) in control (top) and HP-loaded samples (bottom) in Fig 1F,G (*n*=6) showing an increase in G0/G1 phase population and a corresponding decrease in S phase population upon HP (representative experiment from six independent experiments, *n*=263 cells/condition). (I) Representative RNAPII-S2P (magenta) and nuclear (DAPI; blue) immunofluorescence images of cells exposed to cyclic HP for 6 h. (J) Quantification of images in I showing a decrease in RNAPII-S2P intensity in cells exposed to HP (*n*=4 independent experiments with >165 cells/condition/experiment). *P=0.0286 (Mann–Whitney test). (K) qPCR analysis for quiescence and senescent markers in HP loaded and corresponding control samples (*n*=5 independent experiments). **^1^*P*=0.0079, **^2^*P*=0.0079 (Mann–Whitney test). All bar graphs show mean±s.d. g.v., gray values. Scale bars: 50 µm.

To study the effects of physiological levels of cyclic HP on chondrocytes, we constructed a custom pressure chamber (Fig. 1A). Here, a high-performance liquid chromatography (HPLC) pump was combined with a solenoid valve and a pressure sensor to drive cyclic HP with a threshold pressure of 1 MPa. The frequency of the cyclic pressure was controlled to be 0.3 Hz by adjusting flow velocity of the HPLC pump.

To study the effect of HP on chondrocytes, we isolated primary mouse chondrocyte progenitors from femur head articular cartilage, as described in the Materials and Methods. The identity of these cells was validated by morphological analyses as well as key identity genes [Mcam (Cd146), Sox9, Col2A1, Col1A1 and Runx2] and protein (Sox9 and Col2A1) expression (Fig. S1A,B), using the chondrogenic cell line ATDC5 for comparison (Yao and Wang, 2013). The differentiation potential of the isolated chondrocytes into cartilage in response to insulin was also confirmed (Fig. S1C).

To mimic physiological mechanical loading of cartilage, we sealed the chondrocyte-containing culture plates in plastic containers filled with culture medium and deposited them into stainless-steel chambers submerged in a water bath (Fig. 1A). Control cells were subjected to an identical procedure within a non-pressurized water bath. We then exposed cells to cyclic HP at 1 MPa, 0.3 Hz for 6 h and analyzed the effect on chondrocyte state. Analysis of chondrocyte progenitor and differentiation markers revealed that HP increased the protein levels of Sox9, the master regulator of chondrocyte identity (Fig. 1B–D) as well as the chondrocyte identity marker Col2A1 (Fig. S1D) (Lefebvre et al., 2019). In addition, mRNA expression of the progenitor marker Mcam (Li et al., 2011) and markers for immature chondrocytes Acan and Col2A1 were increased (Fig. 1E). In contrast, the mRNA levels for markers for pre-hypertrophic and hypertrophic chondrocytes, as well as osteogenic differentiation, Pthr1, Runx2 and Col1A1, respectively (Lefebvre, 2019), were reduced upon HP (Fig. 1E), suggesting that HP prevents chondrocyte maturation towards the hypertrophic state. Interestingly, the mRNA levels of Sox9 were not altered (Fig. 1E), suggesting that its regulation occurred post-transcriptionally, as has been described previously (Lefebvre, 2019; Lefebvre and Dvir-Ginzberg, 2017).

As the chondrocyte progenitor state is associated with quiescence, whereas the hypertrophic state is characterized by increased proliferation (Li et al., 2017; Wuelling and Vortkamp, 2011), we analyzed proliferation and cycling of these cells in response to HP. Both freshly isolated non-immortalized and SV40-immortalized chondrocytes subjected to 6 h of HP showed reduced incorporation of EdU in a pulse experiment where EdU was applied at the onset of HP (Fig. 1F,G; Fig. S1E). In addition, quantification of DNA content (DAPI intensity) as a function of EdU incorporation revealed an increase in cells in G1/G0 and a concomitant reduction in S phase (Fig. 1H). In contrast, no increase in cell death was observed, as assessed by markers of apoptosis and necrosis (Fig. S1F,G). To further address if HP indeed evoked quiescence or only slowed down replication, we analyzed levels of transcriptional elongation (RNA polymerase II phosphorylated at serine 2; RNAPII-S2P) and observed decreased levels of transcription upon HP (Fig. 1I,J; Fig. S1H). Furthermore, the Sox9-regulated cell cycle inhibitor and stem cell quiescence gene p21 (also known as *CDKN1A*) (Panda et al., 2001) was strongly upregulated in response to HP (Fig. 1K). In contrast, p27 (*CDKN1B*) and p16 (*CDKN2A*), other markers for quiescence/senescence (Diekman et al., 2018; Miller et al., 2007; Zhao et al., 2016), were not induced (Fig. 1K), indicative of a response specific to p21. Collectively these results indicate that cyclic physiological levels of HP induce chondrocyte quiescence and promote the progenitor state.

### Hydrostatic pressure triggers nuclear shrinkage and heterochromatin remodeling

**Fig. 2.**
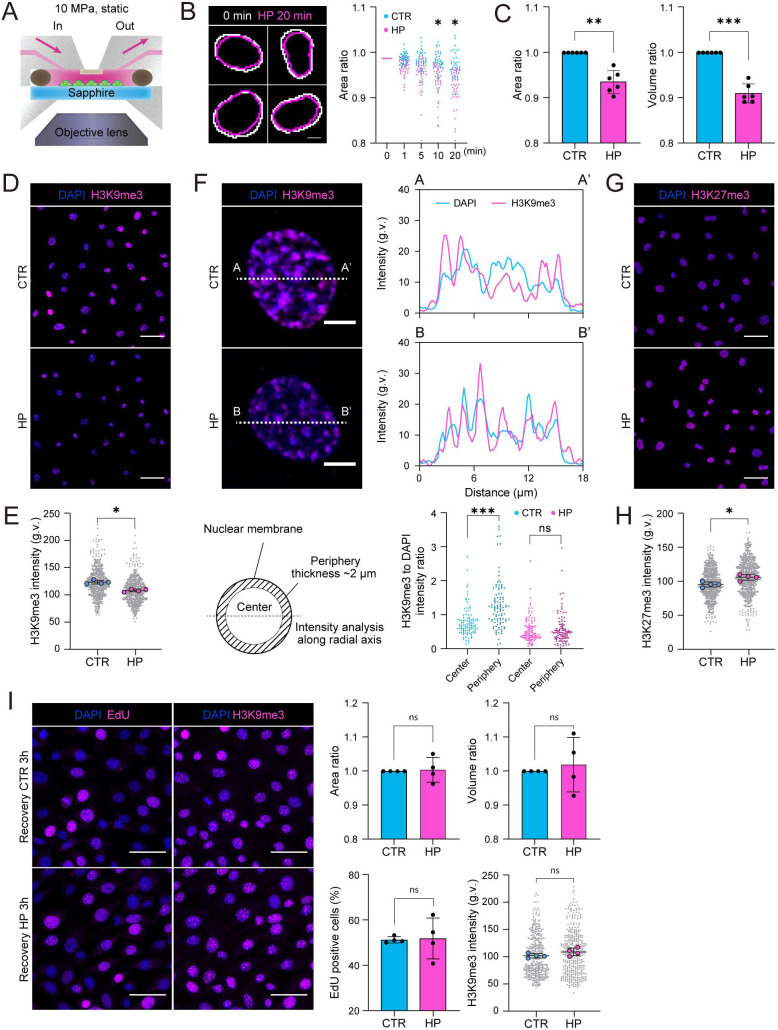
**Hydrostatic pressure triggers nuclear shrinkage and heterochromatin remodeling.** (A) Schematic illustration of the experimental design for live-imaging under HP. Cells seeded on a sapphire glass were treated with Hoechst 33342 to visualize nuclei and loaded under constant HP in a stainless steel chamber mounted on an epifluorescence microscope. (B) Representative nuclear outlines (left) and time-resolved quantification of nuclear area ratio (right) in the absence of loading (cyan) and upon HP (magenta) (*n*=42 cells/condition). *P<0.001 (two-way ANOVA with Sidak's test). Scale bar 5 μm. (C) Quantification of immunofluorescence images showing a decrease in nuclear area and volume in response to cyclic HP, without significant change in nuclear shape (*n*=6 independent experiments with >250 cells/condition/experiment). **P=0.0015, ***P=0.0001 (one sample *t*-test). (D) Representative H3K9me3 (magenta) and nuclear (DAPI; blue) images of cells exposed to cyclic HP for 6 h. (E) Quantification of images in D showing a decrease in H3K9me3 level in response to HP (*n*=4 independent experiments with >130 cells/condition/experiment). *P=0.0286, (Mann–Whitney test). (F) Quantification of H3K9me3 levels within the nuclear periphery and center using linescan analyses (upper panels) and subsequent separation of nuclei into two areas of interest (lower panels). Intensity ratio (H3K9me3:DAPI) within central and peripheral areas of interest showing decreased H3K9me3 in particular at the nuclear periphery upon HP (*n*=100 cells/condition). ***P<0.0001 (Friedman/Dunn's test). (G) Representative H3K27me3 (magenta) and nuclear (DAPI; blue) images of cells exposed to cyclic HP for 6 h. (H) Quantification of images in G showing increased H3K27me3 levels upon HP (*n*=4 independent experiments with >115 cells/condition/experiment). *P=0.0286 (Mann–Whitney test). (I) Representative EdU and H3K9me3 (magenta) and nuclear (DAPI; blue) images of chondrocytes exposed to cyclic HP for 6 h followed by 3 h incubation. Intensity and nuclear size quantifications in bar graphs show no significant differences (*n*=4 independent experiments with >80 cells/condition/experiment). *P*>0.05 (ns, not significant) for all analysis (Mann–Whitney test). All bar graphs show mean±s.d. g.v., gray values. Scale bars: 5 μm (B,F), 50 μm (D,G,I).

To understand how HP regulates chondrocyte identity and state, we sought to identify immediate cellular effects of HP. To this end, we established a unique experimental setup to facilitate live imaging of cells under HP (Fig. 2A). Here, ATDC5 chondrocytes were seeded on a transparent rigid sapphire glass substrate to minimize focus change induced by substrate deformation upon HP. The sapphire substrate was placed in a stainless steel chamber and chondrocytes stained with Hoechst 33342, to visualize nuclei, were exposed to constant HP of 10 MPa under live imaging. The most obvious immediate response to HP was the decrease in nuclear size, which occurred within minutes of HP application (Fig. 2B; Movies 1 and 2). Decreased nuclear area and volume were confirmed by immunofluorescence analyses of fixed mouse chondrocytes (Fig. 2C). Interestingly, no significant alterations in nuclear circularity were observed (Fig. S2A). Although, as expected, nuclear volume was influenced by cell cycle (Neumann and Nurse, 2007), the HP-induced decrease in nuclear size was independent of EdU incorporation levels (Fig. S2B), suggesting that the effect of HP on replication/quiescence did not explain the reduced nuclear volume.

To understand the mechanisms of the nuclear volume decrease, we analyzed the nuclear lamina and heterochromatin, which both play important roles in the regulation of nuclear shape and mechanics (Kirby and Lammerding, 2018; Miroshnikova et al., 2017). Lamin A/C levels were not significantly altered in response to HP (Fig. S2C), consistent with previous studies showing that lamin A levels scale with tensile stiffness (Swift et al., 2013) rather than bulk compression as applied here. In contrast, we observed that the levels of constitutive, H3K9me3-marked heterochromatin were reduced (Fig. 2D,E; Fig. S2D). This reduction was particularly evident at the nuclear periphery, proximal to the lamina (Fig. 2F). In contrast, facultative heterochromatin marked by H3K27me3 was found to be increased upon HP (Fig. 2G,H; Fig. S2E). As H3K9me3-marked heterochromatin is considered more compacted than H3K27me3, we hypothesized that the net effect of this HP-induced chromatin remodeling would be chromatin decompaction. Indeed, analysis of bulk chromatin accessibility using micrococcal nuclease (MNase) digestion revealed a small but reproducible increase in chromatin accessibility to MNase (Fig. S2F). Interestingly, the HP-induced changes in nuclear size, H3K9me3 heterochromatin and EdU incorporation were restored to steady state within 3 h after halting pressurization, suggesting that the responses are reversible (Fig. 2I), similar to what has been described for osmotic stress (Irianto et al., 2013). Together, these data indicate that HP triggers reversible nuclear shrinkage and a reduction in H3K9me3 particularly at the nuclear periphery with a parallel increase in H3K27me3.

### H3K9me3 heterochromatin regulates chondrocyte quiescence and identity

**Fig. 3.**
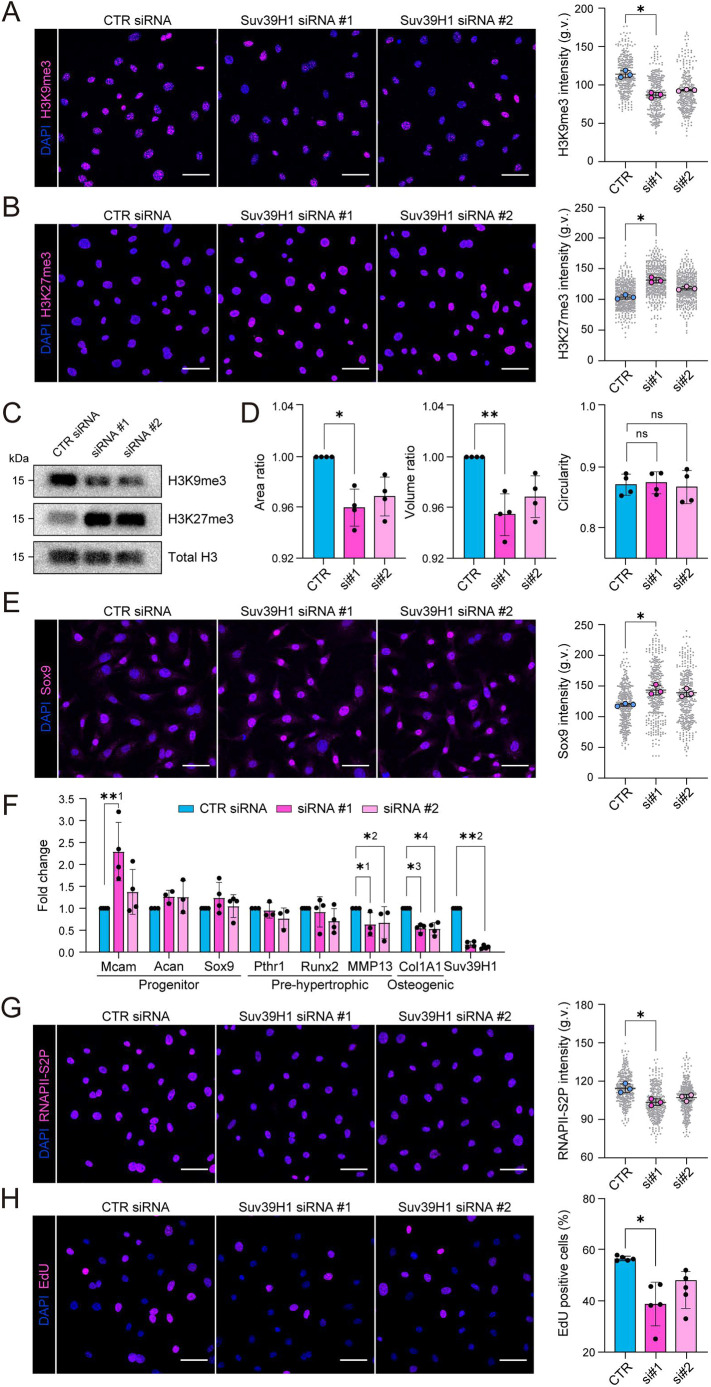
**H3K9me3 heterochromatin regulates chondrocyte quiescence and identity.** (A) Representative H3K9me3 (magenta) and nuclear (DAPI; blue) immunofluorescence images of cells upon siRNA knockdown of Suv39H1 (left panel). Right panel shows quantification of H3K9me3 intensity (*n*=3 independent experiments with >100 cells/condition/experiment). *P=0.0286 (Friedman/Dunn's test). (B) Representative H3K27me3 immunofluorescence images upon Suv39H1 knockdown and intensity quantification (*n*=3 independent experiments with >110 cells/condition/experiment). *P=0.0286 (Friedman/Dunn's test). (C) Representative western blots for histone modification H3K9me3 and H3K27me3 upon Suv39H1 knockdown. (D) Quantification of nuclear area, volume and circularity (*n*=4 independent experiments with >110 cells/condition/experiment). *P=0.0228, **P=0.0094, ns, not significant (Friedman/Dunn's test). (E) Representative Sox9 immunofluorescence images and intensity quantifications of in control and Suv39H1 knockdown cells (*n*=3 independent experiments with >110 cells/condition/experiment). *P=0.0286 (Friedman/Dunn's test). (F) qPCR analysis for chondrocyte progenitor markers Mcam, Acan and Sox9, and pre-hypertrophic and osteogenic differentiation markers Pthr1, Runx2, MMP13 and Col1A1 in Suv39H1 knockdown samples (*n*=3 or 4 independent experiments). *^1^*P*=0.0487, *^2^*P*=0.0338, *^3^*P*=0.0166, *^4^*P*=0.0166, **^1^*P*=0.0095, **^2^*P*=0.0038, (Kruskal–Wallis/Dunn's test). (G,H) Representative RNAPII-S2p and EdU immunofluorescence images and intensity quantifications of in control and Suv39H1 knockdown cells [*n*=3 (RNAPII-S2P) or 5 (EdU) independent experiments with >110 cells/condition/experiment]. *P=0.0286 (RNAPII-S2p); *P=0.0228 (EdU) (Friedman/Dunn's test). All bar graphs show mean±s.d. g.v., gray values. Scale bars: 50 µm.

To understand whether the observed decrease in H3K9me3 and increase in H3K27me3 contributed to the effects of HP on chondrocyte state, we depleted the methyltransferases responsible for catalyzing these histone modifications using siRNA. Depletion of the H3K9me3 methyltransferase Suv39H1 led to an expected decrease in H3K9me3 levels (Fig. 3A). As reported previously in other cell types (Cooper et al., 2014; Le et al., 2016; Peters et al., 2003), the decrease in H3K9me3 was accompanied by an increase in H3K27me3 (Fig. 3B,C). Interestingly, depletion of Suv39H1 and the subsequent reduced H3K9me3 was sufficient to trigger a decrease in nuclear volume without affecting circularity (Fig. 3D), a similar morphological change in the nucleus to that observed in response to HP. Suv39H1 depletion also resulted in increased Sox9 protein expression (Fig. 3E). Similar to what was seen with HP, Sox9 mRNA levels were not significantly altered, whereas the levels of Mcam and to a lesser extent Acan, were slightly increased. More prominently, markers for hypertrophic maturation (Pthr1, MMP13 and Col1a1) were decreased (Fig. 3F). Furthermore, we observed reduced transcriptional elongation (RNAPII-S2P) and EdU incorporation in response to Suv39H1 depletion (Fig. 3G,H). Thus, Suv39H1 depletion to a large extent phenocopies the effects of HP on chondrocytes, attenuating maturation towards the hypertrophic chondrocyte state while decreasing nuclear volume, replication and transcription.

Depletion of the H3K27me3 methyltransferase Ezh2 led to a strong decrease in H3K27me3, as expected, but also to an increase in H3K9me3 (Fig. S3A–C). Interestingly, Ezh2 depletion triggered an increase in nuclear volume (Fig. S3D), further suggesting that heterochromatin is capable of modulating nuclear volume. However, in contrast to depletion of Suv39H1, depletion of Ezh2 and the accompanying decrease in H3K27me3 and mild increase in H3K9me3 did not impact Sox9 expression, EdU incorporation or transcription (Fig. S3E,F). Collectively, these experiments suggested that reduced H3K9me3 was responsible for the effects of HP on chondrocyte identity, decreased replication and transcription, whereas increased H3K27me3 might represent a compensatory effect with a possible role in nuclear volume regulation.

### Hydrostatic pressure-controlled chondrocyte quiescence prevents DNA damage

**Fig. 4.**
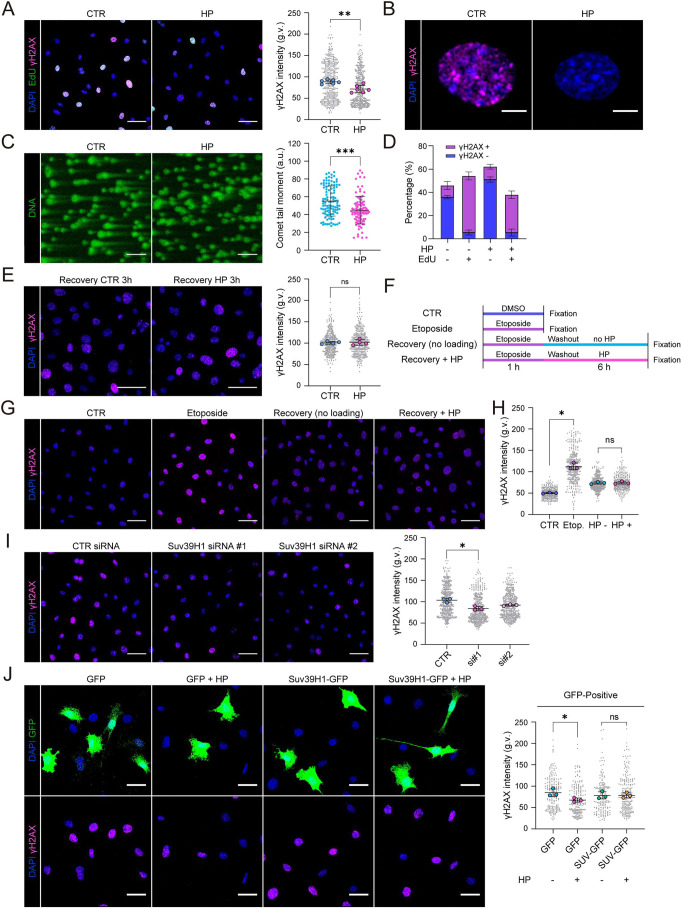
**Hydrostatic pressure-controlled chondrocyte quiescence prevents replicative stress.** (A) Representative EdU (green) and γH2AX (magenta) immunofluorescence images of cells exposed to control (CTR) and cyclic HP for 6 h and intensity quantification of γH2AX showing increased levels of γH2AX upon HP (*n*=6 independent experiments with >250 cells/condition/experiment). **P=0.0050 (paired ratio *t*-test). (B) Representative immunofluorescence image showing pan-nuclear staining pattern of γH2AX in CTR and attenuated signal in HP. (C) Representative images of alkaline comet assays from cells exposed to cyclic HP for 6 h and quantification of comet tails. Note decreased presence of comet tails in cells exposed to HP (*n*=>100 cells/condition). ***P=0.0001 (Mann–Whitney test). (D) Quantification of the relationship of EdU and γH2AX in single cells. Note that EdU-negative cells are predominantly γH2AX-negative, whereas EdU-positive cells are predominantly also γH2AX-positive both in control cells and cells exposed to HP (*n*=3 independent experiments with >180 cells/condition/experiment). (E) Representative γH2AX (magenta) and nuclear (DAPI; blue) images of chondrocytes exposed to cyclic HP for 6 h followed by 3 h incubation (*n*=4 independent experiments with >80 cells/condition/experiment). ns, not significant (Mann–Whitney test). (F) Schematics of the recovery experiment with HP loading after DNA damage induction by pulse treatment of Etoposide (10 μM, 1 h). (G) Representative γH2AX immunofluorescence images of cells obtained in the recovery experiment as described in F. (H) Quantification of immunofluorescence images in G showing increased γH2AX signals after the Etoposide pulse but no significant effect of HP loading on removal of γH2AX (*n*=3 independent experiments with >120 cells/condition/experiment). *P=0.0134 (Friedman/Dunn's test). (I) Representative γH2AX immunofluorescence images from cells depleted of Suv39H1 and intensity quantification (*n*=3 independent experiments with >110 cells/condition/experiment). *P=0.0286, (Friedman/Dunn's test). (J) Representative γH2AX immunofluorescence images and quantification from chondrocytes expressing GFP (control) and Suv39H1-IRES-EGFP exposed to cyclic HP for 6 h (*n*=3 independent experiments with >50 cells/condition/experiment) *P=0.0266; ns,not significant (Friedman/Dunn's test). All bar graphs show mean±s.d. a.u., arbitrary units; g.v., gray values. Scale bars: 50 μm (A,C,E,G,I); 5 μm (B); 20 μm (J).

Next, we aimed to understand the functional relationship between the various effects of HP on DNA metabolism – the reduction in H3K9me3 and slight chromatin decompaction, and the coordinated decrease in replication and transcription. The common denominator of these three phenomena is their effect in reducing barriers for transcription and transcription–replication conflicts that lead to replication fork stalling and genomic instability (García-Muse and Aguilera, 2016). As stalled replication forks are sensed by the DNA damage machinery, we thus sought to examine DNA damage and its relationship with replication upon exposure to HP. Immunofluorescence analyses revealed significantly reduced γH2AX (H2AX phosphorylated on S139) in chondrocytes exposed to HP (Fig. 4A,B), whereas control cells showed frequent pan-nuclear staining of γH2AX as an indication of replicative stress (Fig. 4A,B; Fig. S4A,B) (Toledo et al., 2011). Alleviated DNA damage under HP was further verified with an alkaline comet assay (Fig. 4C). γH2AX was particularly high in cells with high EdU incorporation, confirming the relationship between replication and DNA damage (Fig. 4A,D). However, the positive correlation between EdU and γH2AX remained comparable in HP and control cells (Fig. 4D), suggesting that reduced γH2AX upon HP was secondary to reduced replication and transcription, with less opportunity for fork collision. Similar to the responses in nuclear volume, H3K9me3 and EdU incorporation, γH2AX was also restored 3 h after halting pressurization (Fig. 4E).

To test whether exposure to HP indeed reduced generation of DNA damage and did not enhance repair, we induced replicative stress and DNA damage by inhibiting topoisomerase II using etoposide as a short pulse (1 h) (Smart et al., 2008), and, after washing out etoposide, exposed cells to HP (Fig. 4F). Interestingly, no difference was observed between the control and HP on the rate of recovery after DNA damage (Fig. 4G,H), confirming that the initial differences in γH2AX were due to a lesser amount of damage being generated.

To probe the relationship between H3K9me3 and DNA damage, we depleted Suv39H1 and quantified γH2AX intensity. As predicted, Suv39H1-depleted cells displayed lower levels of γH2AX (Fig. 4I). Furthermore, overexpression of Suv39H1-IRES-GFP to prevent decreased H3K9me3 upon HP, also prevented HP from reducing DNA damage (Fig. 4J). Interestingly, Suv39H1 overexpression alone decreased both γH2AX levels (Fig. 4J) and EdU incorporation (Fig. S4C), indicating that H3K9me3 levels have a biphasic effect on replication, where both very high (Suv39H1 overexpression) and very low (Suv39H1 depletion) H3K9me3 levels reduce replication and thereby most likely replication stress. Collectively, these data show that H3K9me3 levels impact replication rates in chondrocytes and that HP-induced chromatin decompaction protects cells against replication stress and DNA damage.

### Loss of quiescence induces replicative stress to promote loss of chondrocyte progenitor state

**Fig. 5.**
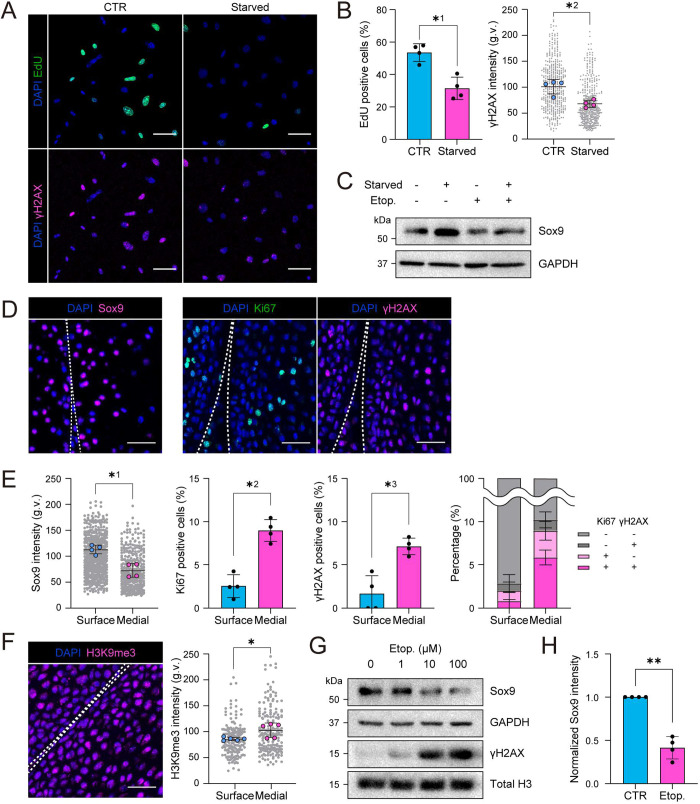
**Loss of quiescence induces replicative stress to promote loss of chondrocyte identity (progenitor state**)**.** (A) Representative EdU/γH2AX chemiluminescence/immunofluorescence images of cells after 24 h serum starvation. (B) Quantification of immunofluorescence images in A showing a decrease in EdU incorporation (top) and γH2AX intensity (bottom) after starvation (*n*=4 independent experiments with >100 cells/condition/experiment). *^1^*P*=0.0286, *^2^*P*=0.0286, (Mann–Whitney test). (C) Representative western blots for Sox9 from cells starved for 24 h followed by Etoposide treatment (10 μM, 6 h). (D) Representative Sox9, Ki67 and γH2AX immunofluorescence images of articular cartilage *in vivo* from postnatal day 2 (P2) mouse cartilage. Note that Ki67-positive cells are predominantly γH2AX-positive in the medial zone. (E) Quantification for Sox9 (left), Ki67 (middle) and γH2AX intensity (right) at surface and medial zones (*n*=4 mice with >90 cells/zone/mouse). *^1^*P*=0.0286, *^2^*P*=0.0286, *^3^*P*=0.0286 (Mann–Whitney test). Distance ranges of 0–30 μm and 30–60 μm from cartilage surface are defined as surface and medial, respectively. Right panel shows the relationship of Ki67/γH2AX in single cells. Note that Ki67-positive cells are predominantly γH2AX-positive in the medial zone (*n*=3 independent experiments with >152 cells/zone/condition). (F) Representative immunofluorescence image and quantification of H3K9me3 in articular cartilage *in vivo* from P2 mice (*n*=5 mice with >90 cells/zone/mouse). *P=0.0317, (Mann–Whitney test). (G) Representative western blots for Sox9 and γH2AX for samples treated with indicated concentrations of Etoposide for 6 h. Note dose-dependent decrease in Sox9. (H) Quantification for Sox9 intensity in western blots for samples exposed to Etoposide treatment (10 μM, 6 h) (*n*=4 independent experiments). **P=0.0028 (one sample *t*-test). All bar graphs show mean±s.d. g.v., gray values. Scale bars: 50 μm (A); 30 μm (D,F).

To dissect the relationship between quiescence, DNA damage and Sox9 expression, we asked whether decreasing replication could reduce replicative stress and thus DNA damage. Promoting quiescence by means of serum starvation reduced both EdU incorporation and γH2AX intensity (Fig. 5A,B). Serum starvation also increased Sox9 expression (Fig. 5C), suggesting that reduced replication might control chondrocyte state. Importantly, a similar correlation was observed in the articular cartilage *in vivo* in postnatal day 2 (P2) mouse cartilage, where the Sox9-positive progenitor cells at the surface zone showed low rates of cycling as defined by Ki67 as well as no γH2AX signal (Fig. 5D,E). In contrast, deeper into the medial zone, the cells had lower Sox9 expression, as expected, and showed higher frequency of Ki67-positive actively cycling cells, which correlated with the γH2AX signal (Fig. 5D,E). Interestingly, and as expected based on high levels of HP, H3K9me3 intensity was lower at the surface zone whereas, deeper into the medial zone, cells showed a higher H3K9me3 intensity, suggesting that physiological stresses *in vivo* in P2 mouse cartilage might trigger heterochromatin changes (Fig. 5F). The levels of H3K27me3 did not, however, substantially differ between the surface and medial zone of the P2 cartilage, indicative of more complex regulation of this histone *in vivo* compared to that in cultured chondrocytes (Fig. S5A).

Finally, we asked if replicative stress/DNA damage could promote chondrocyte differentiation. To this end, we induced DNA damage by inhibiting topoisomerase II using etoposide. Intriguingly, etoposide decreased Sox9 expression in a dose-dependent manner (Fig. 5G,H), suggestive of differentiation.

Taken together, these experiments showed that quiescence associated with the progenitor state of chondrocytes reduces replication stress, whereas promotion of replication stress might trigger chondrocyte differentiation.

## DISCUSSION

Our study demonstrates how hydrostatic pressure regulates chromatin architecture and replication to control chondrocyte differentiation. We observe that cyclic HP mimicking physiological loading of the articular cartilage leads to decreased nuclear volume, chromatin decompaction, and promotion of a quiescent, less mature chondrocyte state, similar to what is found *in vivo* on the surface zone of the articular cartilage.

The observed HP-triggered decrease in nuclear volume is consistent with previous reports where chondrocytes have been subjected to compression and hyperosmotic pressure (Guilak, 1995; Irianto et al., 2013). As in this previous work, the decrease in nuclear volume is an immediate response to HP and is independent of the cell cycle. The decrease in nuclear volume concomitant with chromatin decompaction, however, is somewhat surprising, given that chromatin decompaction is reported to increase nuclear volume through entropic pressure (Mazumder et al., 2008). While this concept is intriguing, it is also reasonable to postulate that the volume changes resulting from chromatin decompaction could be buffered by deformation of nucleocytoplasmic components, water exchange through nuclear pores or other more complex mechanisms. It is also important to consider that HP decreases H3K9me3 particularly at the nuclear lamina, which perturbs the attachment of chromatin to the nuclear lamina (Bondarenko et al., 2017; Towbin et al., 2012), whereas H3K27me3 heterochromatin, which is not anchored to the lamina, is increased. The specific reduction of H3K9me3 at the nuclear periphery might attenuate nuclear membrane tension (Enyedi and Niethammer, 2017; Nava et al., 2020), resulting in decreased volume. As the roles of forces in nuclear volume regulation are not well understood, this aspect remains open for further investigation.

We further observe that HP attenuates maturation of chondrocytes towards a hypertrophic state, characterized by increased expression of immature chondrocyte/progenitor markers Sox9, Acan, Col2A1 and Mcam, and decreased expression of pre-hypertrophic and osteogenic state markers Ptrh1, Runx2 and Col1A1. Effects of HP on chondrocytes have been investigated at various magnitudes, application times and frequencies of cyclic HP. Consistent with our observations, physiological levels of HP (up to 10 MPa) have been reported to increase expression levels of Sox9, Col2A1 and glycosaminoglycans (GAG) in mesenchymal stem cells and cartilage progenitors (Li et al., 2016; Miyanishi et al., 2006), while extreme levels of HP (25 MPa) seem to decrease Col2A1 and Acan expression in a chondrocytic cell line (Montagne et al., 2017). Collectively this implies that there might be a mechanical threshold in chondrocytes, which determines the effect on differentiation. Alternatively, as all the studies use different cell culture models, some of the effects might be cell type specific. More direct comparisons with varying HP amplitudes and frequencies are required to understand how chondrocytes respond to HP under physiological and pathological stress.

We further observe that inducing replication stress decreases Sox9 expression, indicative of increased chondrocyte differentiation. This is consistent with *in vitro* studies of a chondrocytic cell line, where chondrocyte differentiation was associated with increased replication stress (Spaapen et al., 2013). The precise mechanisms by which replication stress could regulate chondrocyte differentiation is unclear. Interestingly, Sox9 has been shown to be targeted for proteasomal degradation upon DNA damage (Hong et al., 2016), and it will be interesting to study whether similar mechanisms operate upon physiological levels of replication stress.

Parallel to the effects on cell state, we observe a widespread reduction in H3K9me3 heterochromatin upon HP, which limits replication and thus replication stress in the chondrocytes. This is consistent with the known susceptibility of heterochromatin regions to replication stress and formation of fragile sites, regions in the genome that show DNA breakage the presence of replication stress (Janssen et al., 2018). A possible explanation of this predisposition of heterochromatin to replication stress could stem from the higher repeat content of heterochromatic regions, resulting in stable secondary structures inhibitory to fork progression (Branzei and Foiani, 2010; Pearson et al., 2005; Zhao et al., 2010) that require additional mechanisms to be properly duplicated (Miller et al., 2006). While not contradictory to these observations, our studies suggest that the degree of chromatin compaction (or phase separation/mobility) could present an additional, sequence-independent factor in promoting susceptibility of heterochromatin to replication stress. This possibility is supported by findings where depleting Suv39H1, to decrease H3K9me3, alleviates excessive DNA damage in progeroid cells and rescues DNA repair upon ATM deficiency (Goodarzi et al., 2008; Liu et al., 2013).

How HP is sensed and how its effects are transduced into the nucleus remain key open questions. As cartilage tissue and chondrocytes are reported to be incompressible under low strains (Bachrach et al., 1998; Ofek et al., 2009), a pressure-sensing mechanism in the absence of a gas–liquid interface remains unclear. One possible mechanism is a change in plasma membrane fluidity, which could activate mechanosensitive ion channels (Nirmalanandhan et al., 2015). A recent study on the endothelial cell cytoskeleton indeed showed that HP-induced cytoskeletal remodeling was mediated by mechanosensitive cation channels and a subsequent elevation in intracellular Ca^2+^ (Prystopiuk et al., 2018). Interestingly, we recently showed that elevated intracellular Ca^2+^, released from the endoplasmic reticulum upon cell stretching or cell compression, mediates a decrease in H3K9me3 (Nava et al., 2020). In addition, intracellular Ca^2+^ is elevated by osmotic swelling of nuclei (Enyedi and Niethammer, 2017). Thus, Ca^2+^ signaling through mechanosensitive channels could be a key mediator of the cellular and nuclear effects in response to a number of stimuli that induce mechanical stress and/or osmotic changes, including stretch, compression and HP.

Taken together, we show that HP acts as a physiological stimulus on chondrocytes that regulates H3K9me3 levels, chromatin architecture and replication to maintain the progenitor state. As the progenitors localize close to the articular surface where pressure is the highest, more in-depth *in vivo* studies will show whether this localization is directly controlled by extrinsic force.

## MATERIALS AND METHODS

### Isolation of murine chondrocytes

Chondrocytes were isolated from femoral heads of postnatal day 20 (P20) male C57BL/6J mice essentially as described previously (Gosset et al., 2008). Femoral heads were carefully isolated, minced and pre-digested in Dulbecco's minimal essential medium (MEM) containing 3 mg/ml collagenase D (Roche) for 30 min. After pre-digestion, cartilage pieces were disrupted by pipetting and digested in Dulbecco's MEM containing 0.5 mg/ml Collagenase D for 1 h. The cell suspension was passed through a 40 μm cell strainer and cells were plated on Mitomycin C-treated (10 µM) J2 fibroblast feeders and cultured in Dulbecco's MEM with 10% fetal bovine serum (FBS; Gibco). After 48 h of cell culture, remaining feeder cells were removed by trypsinization and cells were either used directly for experiments (labeled as non-immortalized cells) or transfected by SV40 large-T antigen for immortalization. Immortalized cells were cultured in Dulbecco's MEM with 10% FBS (Gibco). Non-immortalized cells were used for analyses of quiescence, all other experiments were carried out with immortalized cells. The ATDC5 murine carcinoma-derived chondrogenic cell line was from the ATCC (ECACC99072806) and was cultured in Dulbecco's MEM/Ham's F12 (1:1) with 5% FBS (Gibco). ATDC5 cells were used for initial comparison with isolated mouse chondrocytes and live imaging experiments. All cell culture was performed in 5% CO_2_ at 37°C. All cells were tested negative for mycoplasma. Animals were housed and maintained according to FELASA guidelines in the animal facility of the University of Helsinki. All experiments were approved by local authorities (permit number KEK-18-020).

### Application of cyclic hydrostatic pressure

A total of 300,000 cells (35 mm petri dish) or 30,000 cells (14 mm glass bottom dish) were seeded 24 h prior to the experiment. For pressurization, cell culture dishes were first sealed into a plastic bag filled with culture medium (Dulbecco's MEM with 10% FBS), which were deposited in a stainless steel chamber in a water bath at 37°C. A high-performance liquid chromatography (HPLC) pump (Senshu Scientific) was used to pressurize cells. Frequency of cyclic pressure was controlled by a pressure sensor and a solenoid valve (L. Tex). The threshold of hydrostatic pressure and its frequency was set to be 1 MPa and 0.3 Hz. After HP application was completed, the plastic bag was immediately opened and the cell culture dish was subjected to fixation and lysis for further analysis.

### Real-time imaging of cells under hydrostatic pressure

For real-time imaging, a sapphire glass substrate was coated with fibronectin (10 µg/ml) in phosphate-buffered saline (PBS) for 1 h at 37°C. A total of 30,000 ATDC5 cells were seeded on fibronectin-coated sapphire glass 24 h before the experiment. Cells were treated with Hoechst 33342 for DNA staining 1 h before experiment and then placed in a stainless steel chamber. A high pressure pump (Nihon Seimitsu Kagaku) was used to increase pressure in the chamber, and a relief valve was used to adjust the pressure to be 10 MPa. An epi-fluorescent microscope (IX71; Olympus) with a 30× objective and a cooled CCD (C7780-10; Hamamatsu Photonics) were used for live imaging.

### Transfections, plasmids and RNAi

siRNAs targeting Suv39H1 (#1: s74606, #2: s74607; Thermo Fisher Scientific) and Ezh2 (#1: S65775, #2: S65776; Thermo Fisher Scientific), and negative control siRNA (AM4635) were from Ambion (Silencer Select). Suv39H1-IRES-EGFP was from Sino Biological (ID: 28113). Transfections were performed using Lipofectamine RNAiMax (Thermo Fisher Scientific) for siRNA, and Lipofectamine 2000 (Invitrogen) for protein expression according to manufacturers’ instructions. Experiments were performed after 72 h (siRNA) or 24 h (protein expression) of transfection.

### Immunofluorescence stainings

Cells were fixed in ice-cold methanol for 5 min, permeabilized with 0.3% Triton X-100 in PBS, and blocked in 5% bovine serum albumin (BSA). Samples were incubated overnight at 4°C in primary antibody in 1% BSA and 0.3% Triton X-100 in PBS, followed by washing in PBS and incubation in secondary antibody in 1% BSA and 0.3% Triton X-100 in PBS. Antibodies against the following proteins were used: H3K9me3 (Cell Signaling 13969; 1:5000), H3K27me3 (Cell Signaling 9733; 1:5000), phospho-histone H2A.X (Ser139) (Cell Signaling 9718; 1:5000), phospho-histone H2A.X (Ser139) (Merck Millipore 05-636; 1:1000), lamin A/C (Cell Signaling 4777; 1:5000), Sox9 (Cell Signaling 82630; 1:5000), RNAPII-S2p, (Abcam 5095; 1:5000), collagen 2A1 (Thermo Fisher Scientific MA5-12789; 1:5000), and cleaved caspase-3 (Cell Signaling 9664; 1:1000). The EdU incorporation assay was performed using a Click-iT™ EdU Cell Proliferation Kit (Thermo Fisher Scientific); 1 μM EdU was added to the culture medium filled in the plastic bag prior to HP and detected after fixation.

### Cell viability assay

Cell viability, apoptosis and necrosis was examined with the Apoptotic/Necrotic Cells Detection Kit (PK-CA707-30017; PromoKine), where apoptotic and necrotic cells were labeled with FITC–Annexin V and ethidium homodimer III (EthD-III), respectively. Cells were subjected to HP for 6 h after which samples were subjected to analysis according to the manufacturer's instructions. The stained samples were imaged using an epifluorescence microscope.

### Immunohistochemistry of cartilage

Hind legs of postanal day 2 (P2) C57Bl6 mice were fixed with 4% paraformaldehyde (PFA) for 48 h at room temperature. Fixed samples were decalcified in 10% EDTA (pH 7.4) for 7 days at room temperature on a rocker, and then embedded into paraffin. 8 µm sections were cut with a microtome and deparaffinized, after which antigen retrieval in citrate buffer (pH 6.0) was performed with a microwave (600 W, 30 min). Sections were blocked with 5% goat serum and 3% BSA and subjected to immunofluorescence staining with antibodies against the following: phospho-histone H2A.X (Ser139) (Cell Signaling 9718; 1:500), Sox9 (Cell Signaling 82630; 1:500), Ki67 (Cell Signaling 9449; 1:500), H3K9me3 (Cell Signaling 13969; 1:500) and H3K27me3 (Cell Signaling 9733; 1:500).

### Confocal microscopy

All fluorescence images were obtained by laser scanning confocal microscopy (SP8X; Leica) with Leica Application Suite software (LAS X version 2.0.0.14332), using 40× or 60× immersion objectives. Images were acquired at room temperature using sequential scanning of frames with a step-size of 0.5 μm, after which all planes encompassing complete cell nuclei were projected as a maximum intensity. Images were achieved with the same settings for all samples within an experiment.

### Image analysis

Images were analyzed using Fiji (Schindelin et al., 2012). For imaging of nuclei, fields for observation were randomly selected based on the presence of nuclei assessed by DAPI staining. Confocal stack images were first converted into maximum projection images and nuclear outlines were generated by automated thresholding for DAPI staining and mean fluorescence intensities of nuclear staining of interest were quantified within the nuclear outlines.

For the intensity analysis of periphery and central regions of nuclei, single representative confocal plane with the largest *xy*-cross-sectional area of the nucleus was chosen from confocal stack images (DAPI channel) and the line-scanned intensity profiles for DAPI and H3K9me3 were analyzed. To quantify H3K9me3 intensity in the nuclear periphery versus center, an area of interest was generated by thresholding on maximum projections of DAPI fluorescence. The area of interest was first redirected on midplanes of H3K9me3-stained confocal stacks to quantify mean H3K9me3 intensity (*I*_tot_) in the total nuclear area (*S*_tot_). The area of interest was then reduced by a factor of −11 to shrink it ∼2 µm from the outer edge, and the area was redirected on the midplanes of confocal stacks to quantify mean H3K9me3 intensity (*I*_non_) in the non-peripheral area (*S*_non_). Finally, the peripheral H3K9me3 intensity was calculated by dividing *I*_tot_−*I*_non_ by *S*_tot_−*S*_non._

For nuclear imaging of histone modifications in the femoral articular cartilage from P2 mice, areas of interest were manually generated using the DAPI staining from the contact surface positions (30 µm) towards medial positions (30–60 µm from contact surface) after which mean fluorescence intensities of nuclear stainings were quantified.

### Western blotting

Cells were lysed in the RIPA buffer [150 mM NaCl, 50 mM Tris-HCl, 5 mM EDTA, 0.1% SDS (v/v), 1% sodium deoxycholate (v/v) and 1% Triton X-100 (v/v), supplemented with proteinase inhibitor (cOmplete Mini; Roche) and phosphatase inhibitor (Phos-Stop; Pierce) cocktail tablets] at 4°C and subjected to additional solubilization by sonication using a Bioruptor II (BM Equipment Co., Ltd.) for 15 cycles at 4°C. For histone extraction, cells were first lysed in Triton extraction buffer [PBS containing 0.5% Triton X-100 (v/v), proteinase inhibitor and phosphatase inhibitor] at 4°C, from which nuclei were obtained as a precipitate after centrifugation (2000 ***g*** for 10 min at 4°C). Nuclei were incubated overnight at 4°C in 0.2 M HCl and solubilized histone was collected in a supernatant after centrifugation (17,000 ***g*** for 10 min at 4°C) and neutralized by adding 1.0 M NaOH.

Protein concentrations were quantified with BCA (Thermo Fisher Scientific), suspended in Laemmli sample buffer and denatured. Equal amounts of protein were loaded in 10% SDS-PAGE gels. Peptides separated by SDS-PAGE was transferred to PVDF membrane using the Trans-Blot Turbo Transfer System (Biorad), and the membrane was blocked with 5% BSA or 5% milk in TBS containing 0.2% Tween 20 (TBS-T) for 1 h at room temperature. After blocking, membranes were with primary antibodies overnight at 4°C, washed with TBS-T and incubated with HRP-conjugated secondary antibodies for 30 min at room temperature. HRP was detected by Enhanced Chemi Luminescence (ECL) reaction and chemiluminescence images were obtained using ChemiDoc Imaging System (Bio-Rad). Antibodies against the following proteins were used: GAPDH (Calbiochem 1001; 1:100,000), total H3 (Cell Signaling 3638; 1:10,000), H3K9me3 (Cell Signaling 13969; 1:5000), H3K27me3 (Cell Signaling 9733; 1:5000), phospho-histone H2A.X (Ser139) (Cell Signaling 9718; 1:5000), lamin A/C (Cell Signaling 4777; 1:5000), Sox9 (Cell Signaling 82630; 1:5000), RNAPII-S2p (Abcam 5095; 1:5000), Collagen 2A1 (Thermo Fisher Scientific MA5-12789; 1:5000), and cleaved caspase-3 (Cell Signaling 9664; 1:5000).

### Micrococcal nuclease assay

After pressurization or chemical treatment, cells were washed twice with ice-cold PBS and lysed in lysis buffer (10 mM Tris-HCl pH 7.4, 10 mM NaCl, 3 mM MgCl_2_, 0.5% NP-40, 0.15 mM Spermine and 0.5 mM Spermidine) for 10 min on ice. Nuclei were washed with digestion buffer (10 mM Tris-HCl pH 7.4, 15 mM NaCl, 60 mM KCl, 0.15 mM Spermine amd 0.5 mM Spermidine) and resuspended in 1 mM CaCl_2_-containing digestion buffer. For chromatin digestion, micrococcal nuclease (3 units, New England Biolabs, Inc.) was added to nuclei suspension (100,000 cells in 100 μl) and incubated for 15 min at 37°C. Chromatin digestion was stopped by adding 25 μl of stopping buffer (100 mM EDTA, 10 mM EGTA), followed by protein digestion in 1 mg/ml Proteinase K and 1% SDS (v/v) for overnight at 37°C. Digested genomic DNAs were purified with NucleoSpin Tissue (Macherey-Nagel GmbH & Co. KG) and quantified with a Qubit assay (Thermo Fisher Scientific). Equal amounts of genomic DNAs (25 ng) for each sample was analyzed Agilent 2200 TapeStation with Genomic DNA Screen Tape (Agilent Technologies).

### qPCR

RNA was isolated using the Nucleospin RNA Plus kit (Macherey&Nagel), after which cDNA was synthesized using the High-Capacity cDNA Reverse Transcription Kit (Applied Biosystems). Quantitative (q)PCR was performed on the StepOne Plus Real Time PCR System (Applied Biosystems) using the DyNAmo ColorFlash SYBR Green Mix (Thermo Fisher). Gene expression changes were calculated following normalization to β-tubulin using the comparative Ct (cycle threshold) method. For primer sequences see Table S1.

### Statistical analyses

All experiments performed in the manuscript were repeated at least three times as independent experiments/biological replicates. Sample size was predetermined using power analysis. No datapoints were excluded from the analyses. Statistical analyses were performed using GraphPad Prism software (GraphPad Prism 8). Statistical significance was determined by the specific tests indicated in the corresponding figure legends.

## Supplementary Material

Supplementary information

Reviewer comments
