# Supplementary material for: Hydrostatic pressure prevents chondrocyte differentiation through heterochromatin remodeling
Source: J Cell Sci. 2021 Jan 27;134(2):jcs247643. doi: 10.1242/jcs.247643 (PMC7860130; doi:10.1242/jcs.247643)
Supplement: Supplementary information [file joces-134-247643-s1.pdf]

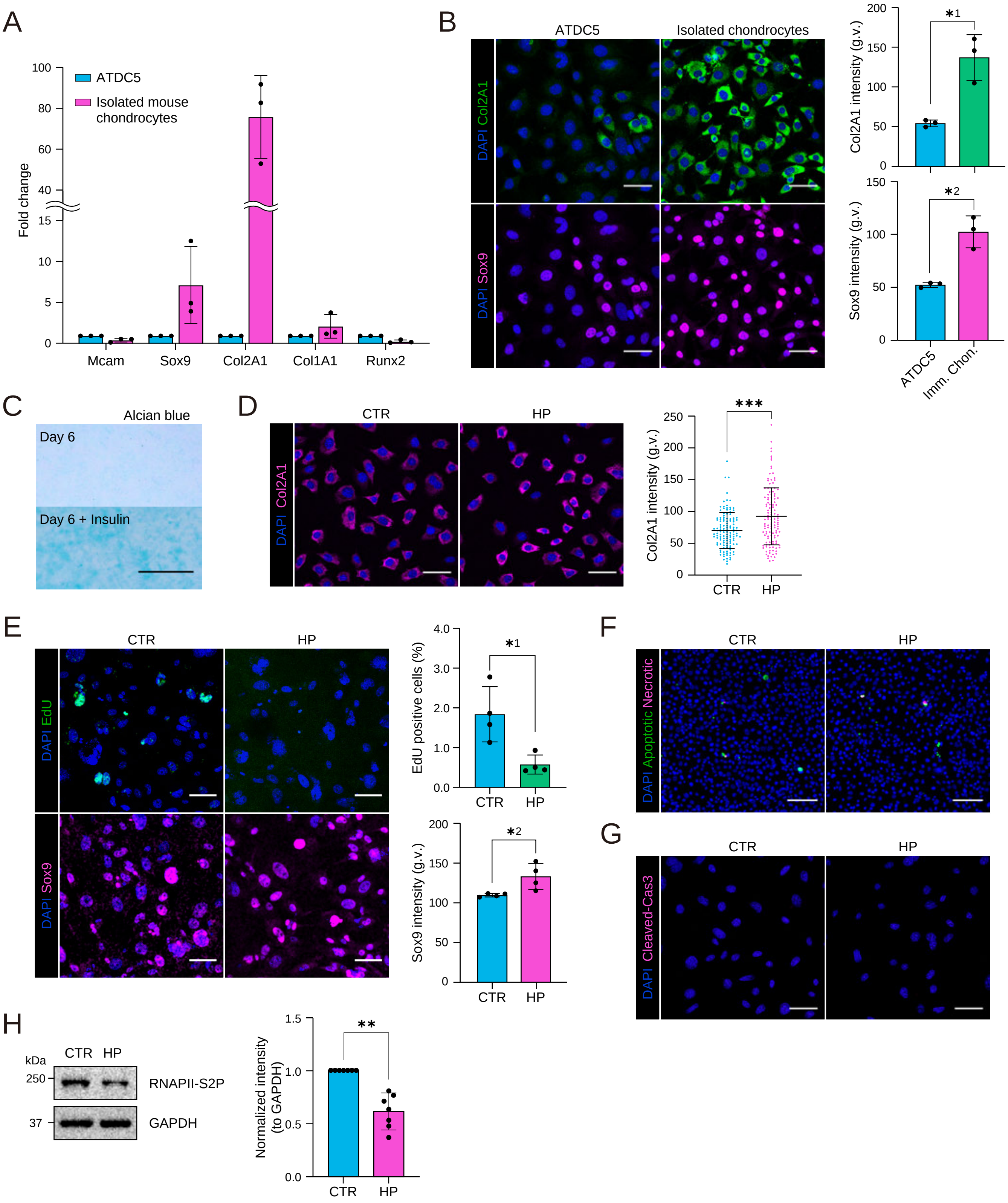

Figure S1.
(A) qPCR of chondrocyte key identity gene expression for ATDC5 and isolated mouse chondrocytes (n = 3 independent experiments). (B) Representative Col2A1 (green), Sox9 (magenta) and DAPI (blue) images of ATDC5 and isolated mouse chondrocytes (n = 3 independent experiments with >155 cells/condition/experiment; $^{*1}p = 0.0280$, $^{*2}p = 0.0326$, Paired t-test). (C) Alcian blue staining for insulin-stimulated mouse chondrocytes. Scale bar 100 μm. (D) Representative Col2A1 (magenta) and nuclear (DAPI; blue) images of chondrocytes exposed to cyclic HP for 6 h (n >110 cells/condition; ***$p < 0.0001$, Mann-Whitney). Scale bar 50 μm. (E) Representative EdU (green), Sox9 (magenta) and nuclear (DAPI; blue) images of non-immortalized chondrocytes exposed to cyclic HP for 6 h (n = 4 independent experiments with >115 cells/condition/experiment; $^{*1}p = 0.0286$, $^{*2}p = 0.0286$, Mann-Whitney. Scale bar 50 μm. (F) Representative FITC-Annexin V (green), Ethidium Homodimer III (magenta) and DAPI (blue) images of cells exposed to cyclic HP. Scale bar 300 μm. (G) Representative Cleaved Caspase-3 (magenta) and DAPI (blue) images of cells exposed to cyclic HP for 6 h. Scale bar 50 μm. (H) Representative western blots and quantification for RNAPII-S2P for samples exposed to cyclic HP for 6 h (n = 7 independent experiments; **$p=0.0012$, One sample t-test). All bar graphs show mean ±SD, g.v. = gray values.

Journal of Cell Science • Supplementary information

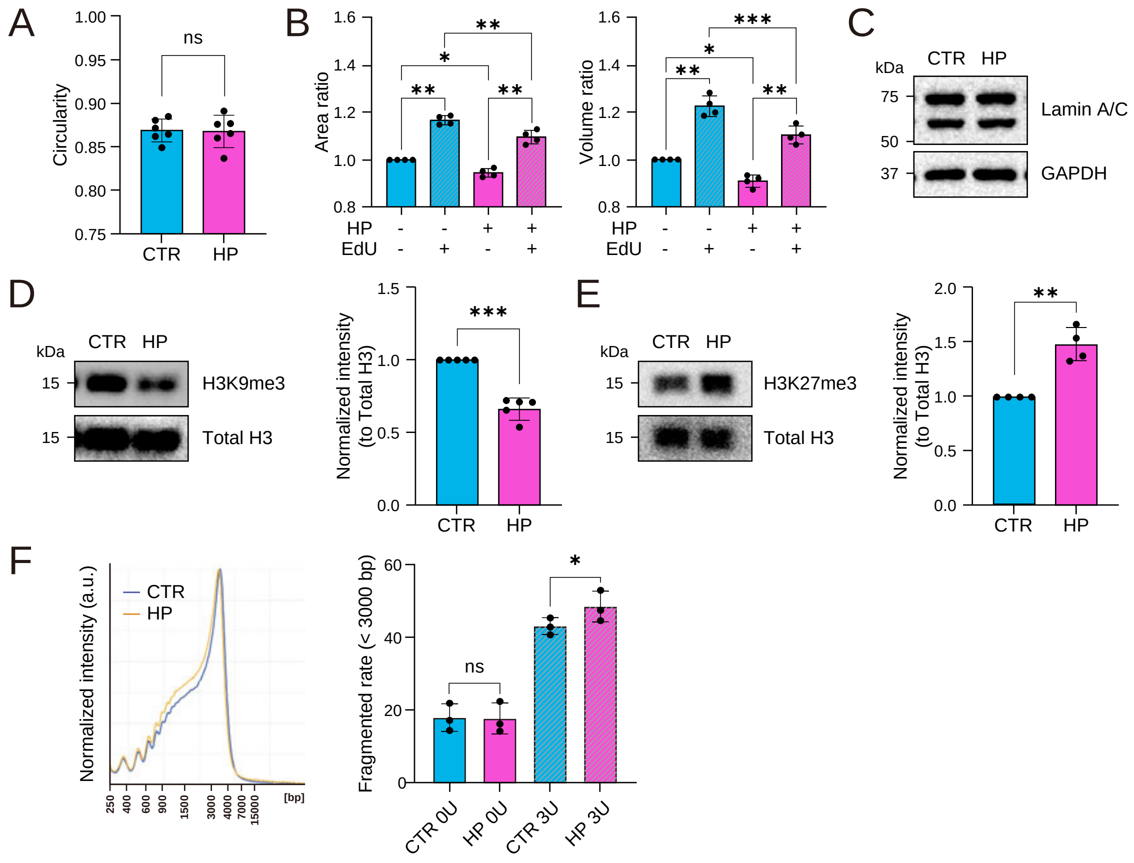

Figure S2.
(A) Quantification of nuclear morphology from DAPI-stained cells (cyclic HP, 6 h) shows no significant change in nucleus circularity (n = 6 independent experiments with >250 cells/condition/experiment). (B) Nuclear area (left) and volume (right) were analyzed for chondrocytes exposed to cyclic HP for 6 h and stained/labeled with DAPI and EdU. Cells were classified into EdU positive and negative groups based on two separated intensity distribution and then subjected to nuclear size analysis (n = 4 independent experiments with >105 cells/condition/experiment; *p < 0.05/**p < 0.01/***p < 0.001, RM one-way ANOVA). (C) Representative western blots and quantification for Lamin A/C from cells exposed to cyclic HP for 6 h. (D, E) Representative western blots and quantification for H3K9me3 and H3K27me3 from cells exposed to cyclic HP for 6 h (n = 5 and 4 independent experiments; ***p = 0.0007 (H3K9me3); **p = 0.0058 (H3K27me3), one sample t-test). (F) Results of Micrococcal chromatin fragmentation assay after HP-loading (left and center panel) (n = 3 independent experiments; *p = 0.0419, ns = not significant, Paired t-test). All bar graphs show mean ±SD.

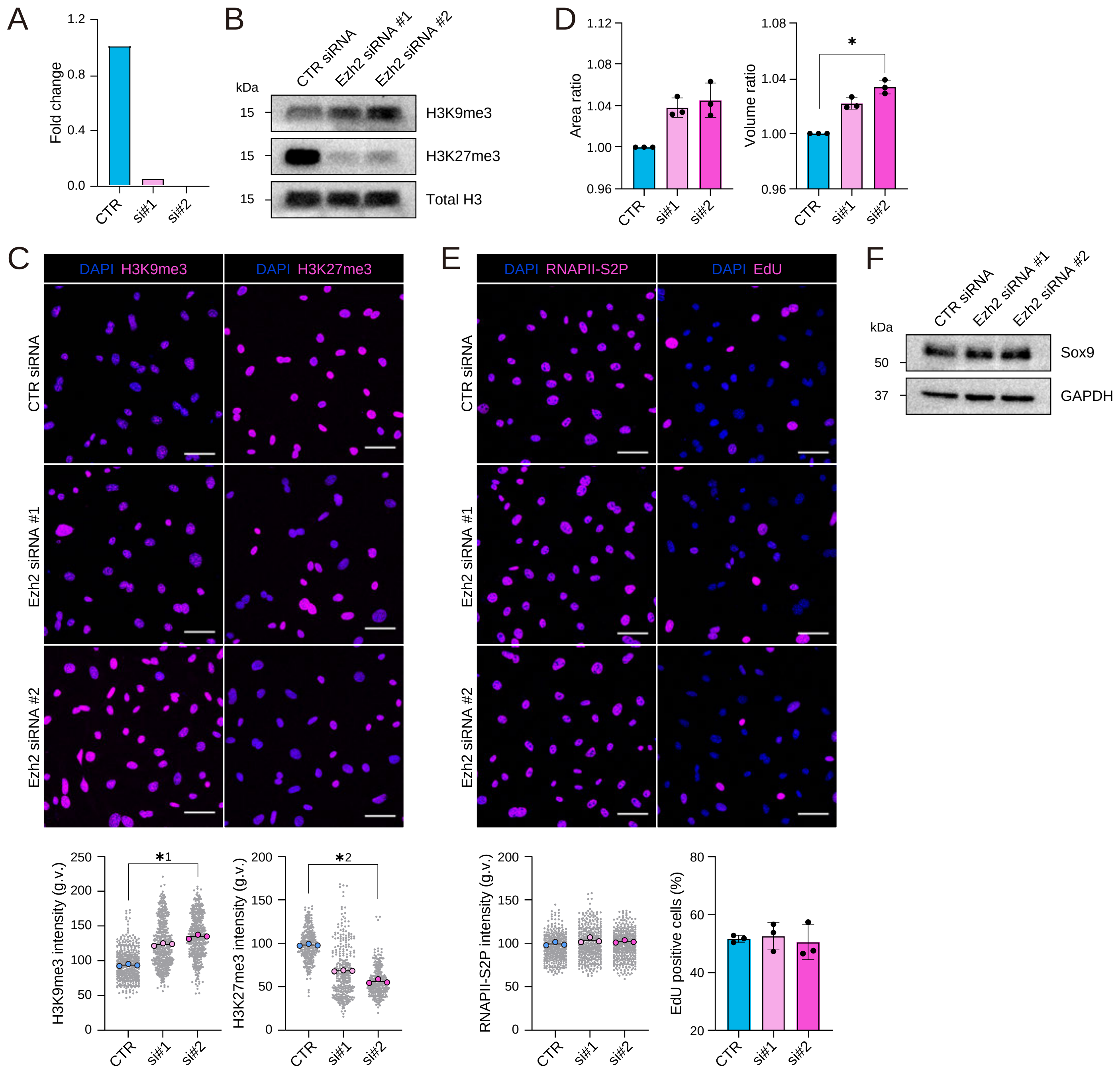

Figure S3.
(A) qPCR for Ezh2 upon siRNA knockdown. (B) Representative western blots for H3K9me3 and H3K27me3 upon Ezh2 knockdown. (C) Representative H3K9me3 and H3K27me3 immunofluorescence images and intensity quantification upon Ezh2 knockdown (n = 3 independent experiments with >105 cells/condition/experiment; $^{*1}p = 0.0286$, $^{*2}p = 0.0286$, Mann-Whitney). (D) Quantification of immunofluorescence images from DAPI-stained cells shows increase in nuclear area and volume in response to HP (n = 3 independent experiments with >105 cells/condition/experiment; $^{*}p = 0.0286$, One sample t-test). (E) Representative H3K9me3, H3K27me3, RNAPII-S2P and EdU immunofluorescence/chemiluminescence images and intensity quantification upon Ezh2 knockdown (n = 3 independent experiments with >110 cells/condition/experiment). (F) Representative western blots for Sox9 upon Ezh2 knockdown. All bar graphs show mean ±SD, scale bars 50 µm, g.v. = gray values.

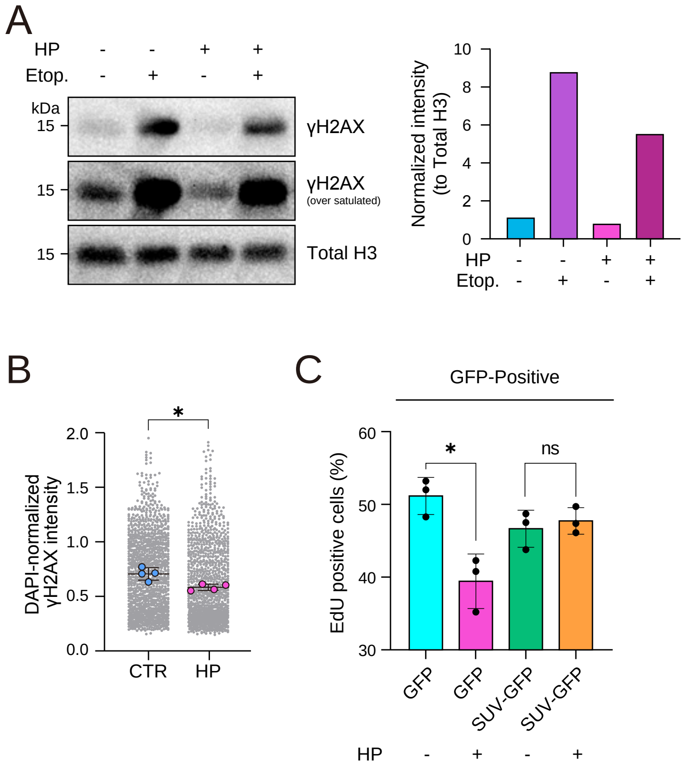

Figure S4.
(A) Representative western blots for γH2AX from chondrocytes exposed to cyclic HP for 6 h with and without etoposide treatment (10 μM). (B) Intensity quantification for γH2AX staining in Fig. 4A by DAPI-normalization shows decreased level of γH2AX upon HP (n = 4 independent experiments with >250 cells/condition/experiment; *p = 0.0286, Mann-Whitney). (C) Quantification of EdU positive cells in chondrocytes expressing GFP (control) and Suv39H1-IRES-EGFP exposed to cyclic HP for 6 h (n = 3 independent experiments with >50 cells/condition/experiment; *p = 0.0266, ns = not significant, Friedman/Dunn's).

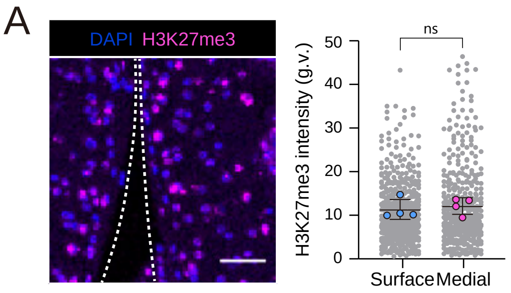

Figure S5.

(A) Representative H3K27me3 immunofluorescence image of articular cartilage from postnatal day 2 (P2) mouse, and quantification of intensity at surface and medial zones (n = 4 mice with >50 cells/zone/mouse; ns = not significant, Mann-Whitney). Scale bar 30 μm, g.v. =,gray values.

Journal of Cell Science • Supplementary information

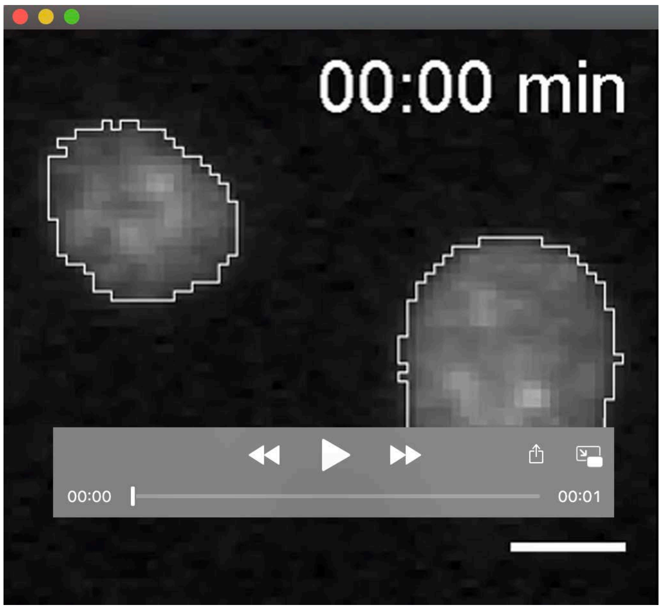

Movie 1. Real time imaging of ATDC5 cells without hydrostatic pressure Representative movie of ATDC cells without hydrostatic pressure. Initial nuclear outline is indicated in a cyan line and the nuclear outline in each frame is traced in magenta. Frame rate 30 sec/frame. Scale bar 20 μm.

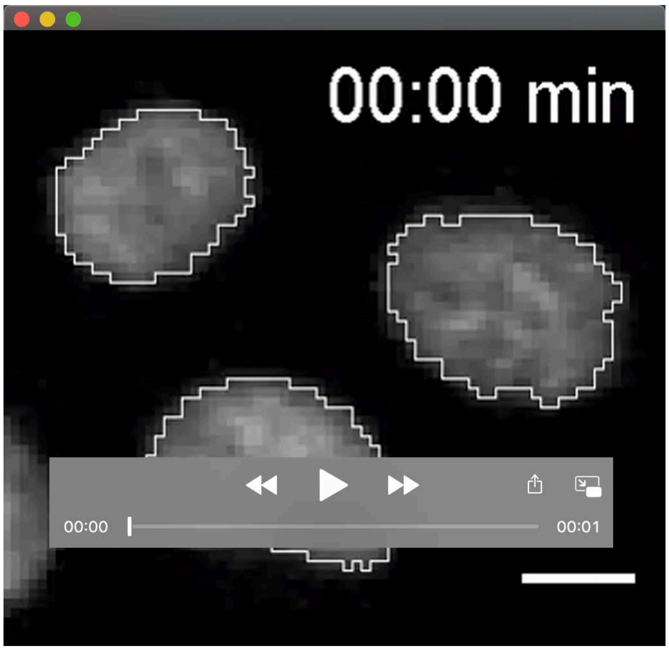

Movie 2. Real time imaging of ATDC5 cells under hydrostatic pressure Representative movie of ATDC cells exposed to 10 MPa hydrostatic pressure. Initial nuclear outline is indicated in a cyan line and the nuclear outline in each frame is traced in magenta. Frame rate 30
sec/frame. Scale bar 20 μm.

Table S1

Gene	Forward	Reverse
Tubb	GAGCAGTTCACAGCCATGTT	CCAGGTCATTCATGTTGCTCTC
Actb	TCAAGATCATTGCTCCTCCTG	TACTTCTGCTTGCTGATCCAC
Col2A1	ACGAAGCGGCTGGCAACCTCA	CCCTCGGCCCTCATCTCTACATCA
Col1A1	GGCTGTGTGCGATGACGTGCAA	TCCGGGCAGAAAGCACAGCACT
Acan1	GTGAGGACCTGGTAGTGCGAGTGA	GAGCCTGGGCGATAGTGGAATATA
Sox9	CGATTACAAGTACCAGCCCCGG	TGCAGCGCCTTGAAGATAGCAT
Suv39H1	TTGGCACCCACTGGAGGCTGTT	TGCCCAGCTTTCAGTCGCACCT
Runx2	ACCCAGCCACCTTTACCTAC	TATGGAGTGCTGCTGGTCTG
Ezh2	TCACGTGTGGAGCTGCTGACCA	AAGATGCCCCAGCCTGCCACAT
Mcam	TGCCATGTGGACGCTCGGGAAA	TCACCGTTGCTGCCCTGAAGGA
P21	TGAGCAGTTGCGCCGTGATTGC	AACGCGCTCCCAGACGAAGTTG
P16	TGCAATCACGGGAGGGAGCAGA	ATTATTCCCTTCGCGGCCGCCT
P27	TCCTGACCAGCGGCTGCATCAT	GCGCCGTAGATCACCGTGTTCT
Pthr1	ACCTTGGCCAACACTGGGTGCT	AGCTTAGTGGCAAGCACCCGGA
MMP13	AGGCGATGAAGACCCCAACCCT	AGGGTGCAGGCGCCAGAAGAAT

Journal of Cell Science • Supplementary information
