# Supplementary material for: Hydrostatic pressure prevents chondrocyte differentiation through heterochromatin remodeling
Source: J Cell Sci. 2021 Jan 27;134(2):jcs247643. doi: 10.1242/jcs.247643 (PMC7860130; doi:10.1242/jcs.247643)
Supplement: Reviewer comments [file joces-134-247643_review_history.pdf]

Journal of Cell Science

Hydrostatic pressure prevents chondrocyte differentiation through heterochromatin remodeling

Koichiro Maki, Michele M. Nava, Clémentine Villeneuve, Minki Chang, Katsuko S. Furukawa, Takashi Ushida and Sara A. Wickström
DOI: 10.1242/jcs.247643

Editor: Andrew Ewald

Review timeline

Original submission:	15 April 2020
Editorial decision:	4 June 2020
First revision received:	11 September 2020
Editorial decision:	21 October 2020
Second revision received:	9 November 2020
Accepted:	1 December 2020

Original submission

<u>First decision letter</u>

MS ID#: JOCES/2020/247643

MS TITLE: Hydrostatic pressure promotes chondrocyte quiescence and progenitor state through heterochromatin remodeling and suppression of replicative stress

AUTHORS: Koichiro Maki, Michele M Nava, Clémentine Villeneuve, Minki Chang, Katsuko S Furukawa, Takashi Ushida, and Sara A Wickström
ARTICLE TYPE: Research Article

We have now reached a decision on the above manuscript.

To see the reviewers' reports and a copy of this decision letter, please go to: https://submit-jcs.biologists.org and click on the 'Manuscripts with Decisions' queue in the Author Area. (Corresponding author only has access to reviews.)

As you will see, the reviewers are unanimous in their appreciation that this is an important problem and that the conclusions are exciting. However, they each identify a different set of issues in terms of the details of the support for these conclusions. They suggest that a revised version might prove acceptable, if you can address their concerns. If you think that you can deal satisfactorily with the criticisms on revision, I would be pleased to see a revised manuscript. We would then return it to the reviewers.

We are aware that you may currently be unable to access the lab to undertake experimental revisions. If it would be helpful, we encourage you to contact us to discuss your revision in greater detail. Please send us a point-by-point response indicating where you are able to address concerns raised (either experimentally or by changes to the text) and where you will not be able to do so within the normal timeframe of a revision. We will then provide further guidance. Please also note that we are happy to extend revision timeframes as necessary.

Please ensure that you clearly highlight all changes made in the revised manuscript. Please avoid using 'Tracked changes' in Word files as these are lost in PDF conversion.

© 2020. Published by The Company of Biologists under the terms of the Creative Commons Attribution License (https://creativecommons.org/licenses/by/4.0/).

I should be grateful if you would also provide a point-by-point response detailing how you have dealt with the points raised by the reviewers in the 'Response to Reviewers' box. Please attend to all of the reviewers' comments. If you do not agree with any of their criticisms or suggestions please explain clearly why this is so.

Reviewer 1

Advance summary and potential significance to field

Understanding cell fate changes under pressure can be important to multiple tissues, including cartilage studied here.

Comments for the author

The authors describe the effects of high pressure on chondrocyte fate, which is certainly relevant to cartilage as tissue pressures can be high. Some aspects seem novel, but the study seems lacking in several ways, which tempers enthusiasm:

1. First, please rule out potential artifacts: Does pressure impede staining for DNA or antibody entry for immunostaining? Does pressure impede EdU reactivity?

2. For Fig.1: has Sox9 immunostaining been verified by immunoblot?
The increase in Col2A1 mRNA is only ~50%; is that relfected in protein change and is it biologically significant? Does nuclear area vary with cell cycle stage (as relevant to Fig.1F,G and 2B,C?

3. High hydrostatic pressure in cartilage is balanced by hyperosmotic pressure, which is a better way to say with "high water content chondrocytes within the cartilage are exposed to high levels of hydrostatic pressure". Importantly, for chondrocytes, hyperosmotic pressure indeed has a similar effect as here "Hydrostatic pressure triggers nuclear shrinkage" as previously quantified for hyperosmotic stress in Irianto (Biophys J 2013)
- as should be cited. The same paper also provided EM and additional evidence that 'Osmotic Challenge Drives Rapid and Reversible Chromatin Condensation in Chondrocytes'. Kinetics and reversibility were clarified in that paper; are the changes here similarly reversible and rapid?

4. Fig.3 is not especially novel: Peters (Cell 2001) showed in mice that Loss of the Suv39h Histone Methyltransferases Impairs Mammalian Heterochromatin and Genome Stability.

5. Fig.4A,4B,5B: is gH2AX verified by immunoblot (to avoid pressure artifacts on staining)? Normalize gH2AX by DNA per nucleus to determine whether the DNA damage density is affected by pressure.

6. Fig.4I: Does overexpression of Suv39H1 fully rescue High Pressure effects? If partial rescue, then why?

7. The authors state that "Lamin A/C levels were not significantly altered in response to HP (Supplementary Fig. 2B)". It should be cited that Lamin A/C has been previously reported to correlate with and respond to tensile stiffness (Swift 2013) rather than bulk compression as applied here.

8. I have missed evidence for the statement: "promoting replicative stress by inhibition of topoisomerase II triggers chondrocyte differentiation". Knockdown of Topo-II would also seem necessary.

Minor:
1. Statistical significance needs to be scrutinized for bars with overlapping errors, including Fig.2A,4C,S2E.

© 2020. Published by The Company of Biologists under the terms of the Creative Commons Attribution License (https://creativecommons.org/licenses/by/4.0/).

Reviewer 2

Advance summary and potential significance to field

This study by the Wickstrom lab utilizes several bespoke platforms for applying differing levels of hydrostatic pressure to chondrocytes. They show that high hydrostatic pressure (HP) promotes chondrocyte quiescence and identity. They show this state is at least associated with chromatin decompaction and increase in H3K27me3-marked facultative heterochromatin, and induced by a reduction in H3K9me3-marked constitutive heterochromatin. They finish by showing that the main mechanism for the maintenance of the chondrocyte progentitor state is reduction of replicative stress, associated with a reduction of H3K9me3.

This is a clear, interesting, and important paper that continues along a research thread, exemplified by the Wickstrom lab, showing a relationship between mechanical stress and chromatin modifications that mitigate DNA damage and affect transcription in progenitor cells. I have a number of comments, but, importantly, they are minor, and aside from potentially a quantification in point 1 and perhaps a small experiment in point 2, I would not expect any of them to require additional experiments.

Comments for the author

1. I'm a little uncomfortable with the implication that overall higher levels of Sox9+ in a cell would make it a better progenitor cell. At the least, it might be interesting to supplement the data in Fig. 1C with a quantification of + and 1, binning in thirds, for example, and examining how many + and – cells there are (using the bottom and top third, for example). I just don't know what it means to express Sox9 more in a cell that already expresses it, when TFs could potentially just be in excess at some point.
2. It should be checked whether apoptosis or live/dead ratio is changed upon HP.
3. I'm a little confused about the role of chromatin decompaction here. Why would this correlate with a loss in nuclear size? Maybe I'm not sufficiently up on the literature, but doesn't it seem it should be the opposite? You're increasing the entropy which should relieve the entropic pressure, at least locally. If you are applying an external pressure that should more than compensate, but the Suv39h1 depletion leading to loss of nuclear size is surprising to me because there's no compensating HP. To me this is a genuinely surprising result worthy of further comment, if possible. Same goes for role of chromatin compaction (via H3K27me) in size increase.
4. It's really not clear that H3K27me3 increases are compensatory. The data on the role of Ezh2/H3K27me3 are not super conclusive and the feedback between these two methylation marks is not understood all that well. I'm not a connoisseur so I could be wrong and I'm happy to be corrected on this if so. Moreover, the specificity of these enzymes, in particular Suv39h1, is pretty high, but it's unclear, at least to me, whether or not other enzymes could play a compensatory role upon depletion of the named ones. Could the authors comment? Words like 'responsible' are used without modifiers so I just want to make sure this is warranted by the data. Also, not sure about statement 'whereas increased H3K27me3 likely represented a compensatory effect with a possible role in nuclear volume regulation' – why would it have a role in nuclear volume regulation? This is similar to point 3. There could be quite a bit going on here.
5. It might be good to scale back conclusions about replicative stress leading to differentiation. This seems strange (as acknowledged in Discussion) and the data is not highly conclusive without more support. I wouldn't recommend pushing the point further, it's an interesting observation, but the language should reflect that. Simply using words like 'suggest' as opposed to 'show' helps, and more of an acknowledgement in conclusions about potential weaknesses in current data for that.
6. On that same point, there are a few examples where the authors are verging on overstatement based on the data. It is not blatant, but given that the data are extremely interesting in and of themselves and will represent an important contribution to the literature it would be good to be extra careful about this. An example is the line 'To dissect the possible causative relationship between quiescence, replicative stress and chondrocyte identity, we asked if decreasing replication could reduce replicative stress/DNA damage'. I don't think that serum starvation and Fig. 5D, E are showing anything causative since serum starvation is a blunt tool and Fig. 5D, E are correlative.

© 2020. Published by The Company of Biologists under the terms of the Creative Commons Attribution License (https://creativecommons.org/licenses/by/4.0/).

7. Not sure what ‘Thus, Ca2+ signaling is through mechanosensitive channels is a likely key mediator of the cellular and nuclear effects of a number of extrinsic forces, including stretch, compression, and HP, suggesting existence of a generic mechanoresponse across various extrinsic forces.’ means. Could the authors clarify?
8. It’s at least implied at several points that mechanical stress is causing the changes seen. HP certainly increases mechanical stress, but it could be the volume changes themselves that lead to the changes, as one would see with increasing osmolarity. So in this sense, the effect of mechanical stress is indirect. Granted, it doesn’t matter that much in the grand scheme of things, but I think it’s important to be precise on this point particularly in the discussion.

Reviewer 3

Advance summary and potential significance to field

How mechanoregulation of chondrocyte differentiation states and the associated molecular mechanisms impact on chondrocyte fate is an important biological question relevant to cartilage development and skeletal diseases.
Hydrostatic pressure is considered as one of the most important mechanical stimuli for cartilage. In this paper Maki K. et al aim to provide molecular insight into the mechanism how mechanical loading affects chondrocyte differentiation and maintenance. They investigated the impact of hydrostatic pressure (exerted via a custom made pressure chamber) on chromatin modification states of isolated primary chondrocytes and the ATDC5 chondrogenic cell line. Quantitative measurements were made of nuclear changes by live imaging experiments. The role of changes in histone modification on chondrocytes was assessed by investigating the impact of manipulating histone modifications by depleting Suv39H1 H3K9me 3 methyl transferase using siRNA.
The association of histone modification changes with replicative stress and the impact on chondrocyte cell state and differentiation was also studied.
They conclude that mechanical stimuli promotes chondrocyte quiescence and progenitor state via inducing chromatin decompaction and reducing replicative stress. In general the quality of the data is high and the paper well
written. However there are a number of major issues which limit the scope of the bold interpretation and conclusions regarding the mechanistic link invoking heterochromatin remodeling, replicative stress and chondrocyte quiescence and progenitor state as stated in the title.

Comments for the author

Major concerns
1. There are several major caveats limiting the interpretation of the data and in vivo relevance of the findings. The first caveat is related to the chondrocyte population studied. Assessments of changes in gene expression were done by qRT-PCR. However this is limited by the fact that it is likely that the starting population of chondrocytes isolated from articular cartilage will be heterogeneous in their differentiation state, there are possibly perichondrium cells present and additionally the cells are transformed by SV40 T antigen which will impact on gene expression and their chondrocyte identity.
In addition, cells in the surface and medial zones of articular cartilage may respond differently to the mechanical loading. Sub-fractionation of the starting chondrocytes may be an option, or validating the observed changes in heterochromatin and the expression of progenitor markers in intact articular cartilage. An additional question arising is to what extent does the SV40 T antigen immortalization induce the reactivation of DNA synthesis in the nuclei of non-dividing articular chondrocytes (PMID: 2548682)? This is relevant to the Edu and DNA replication experiments.
2. Using qRTPCR for Sox9, Acan1 and Col2a1 alone as proof of chondrocyte identity is not sufficient. Validation of qRTPCR results should be done by immunostaining/ in situs on the cells themselves. Additional assessments for genes marking different differentiation states (proliferating, hypertrophic), cell cycle status and testing for the expression not characteristic of chondrocytes such as Col1a1 should be included. The data presented in Supplementary Fig.1 – alcian blue staining lack cellular resolution and hence hetereogeneity in gene expression cannot be assessed. Staining for SOX9 and other markers would be more convincing to show the chondrocyte identity of the cells. In Fig.1 B it appears that there are more SOX9 expressing cells in the HP sample and the

© 2020. Published by The Company of Biologists under the terms of the Creative Commons Attribution License (https://creativecommons.org/licenses/by/4.0/).

intensity in individual cells varies. It would be informative to quantify the number of SOX9 positive cells.
3. SOX9 is also known to promote chondrocyte proliferation, but there seems to be a dichotomy between number of Edu cells and number of SOX9 expressing cells in Fig, 3 E-G. Please explain if transcription is down and SOX9 expression
(immunostaining) is upregulated – is this effect at translation/protein stability or transcription. The impact of Suv39H1 knockdown on the number of SOX9 expressing cells and on chondrocyte identity/differentiation status and quiescent state should be shown using molecular markers.
4. As in vivo cartilage is exposed to stresses between 0.2 – 10 MPa, do the cells respond differently to different levels of pressure? In this study the hydrostatic pressure in the study is stated as set to 1 MPa. There should be discussion comparing this work with a previous study on ATDC5 where changes in the expression of Polycystins, SOX9, and RUNX2 were found in response to constant hydrostatic pressure of 14.7 kPa (PMID: 27909759).
5. A major omission of the analyses is the inclusion of indicators of established mechanosensitive pathways, such as MRTF/SRF and Hippo signalling 6. A major focus of the paper is to correlate the changes in chromatin modification induced by HP with transcription, replication and DNA damage and a quiescence state of the chondrocytes. What is the molecular basis of concluding that the chondrocytes are quiescent? Is this because there is nuclear shrinkage? Is it possible that the chondrocytes have entered a senescent state in response to HP? It would be important to define the quiescent versus senescent state by molecular markers (see e.g. PMID:
29044508)
7. The logic of concluding that reduced γH2AX in HP was secondary to reduced transcription and replication (page 6) is not clear. Reduced Edu incorporation should reflect reduced replication and therefore less replicative stress?
8. Concerns about data presentation:
Figure 4A the levels of γH2AX are decreased upon HP not increased.
In Figure 4B, which is CTR and HP?
In Figure 4I, the intensity of γH2AX in SUV-GFP with HP condition is higher than SUV-GFP without HP. Does it mean SUV overexpression and HP condition has a negative feedback effect?
In Figure 5D, is it possible to show the chromatin state (H3K9me3, H3K27me3)
in in vivo articular cartilage?
9. The results obtained in this study should be discussed in the context of the other studies on the hydrostatic pressure in chondrocytes, such as PMID:
28813497, PMID: 27909759, PMID: 28085114, PMID: 26528971. How do the changes in chromatin compare with early studies on chromatin rearrangement during chondrogenesis and C-heterochromatin distribution (PMID: 1927209).
Minor issues 1. Clarification about quantitation: How the normalization of results shown in the figures was done should be stated. For example how was normalized intensity determined? Examples are the Y axis in the bar charts such as Figure 1C, Figure 2C, 2H etc.
2. Inaccurate statements such description of the process of endochondral ossification. There are many publications that show that chondrocytes are now known to survive to become osteoblasts in bone formation (reviewed in PMID:
30949840)
3. The sentence on page 6 “immunofluorescence analyses revealed significantly reduced upon γH2AX …” doesn’t make sense.
4. On page 8 the sentence “As cells are essentially aqueous liquids….” is obviously not accurate.

First revision

Author response to reviewers' comments

Reviewer 1

The authors describe the effects of high pressure on chondrocyte fate, which is certainly relevant to cartilage as tissue pressures can be high. Some aspects seem novel, but the study seems lacking in several ways, which tempers enthusiasm:

© 2020. Published by The Company of Biologists under the terms of the Creative Commons Attribution License (https://creativecommons.org/licenses/by/4.0/).

We thank the reviewer for the positive assessment of the relevance of our study and the expert feedback that helped us to further strengthen it.

1. First, please rule out potential artifacts: Does pressure impede staining for DNA or antibody entry for immunostaining? Does pressure impede EdU reactivity?

We agree that this is an important point and thus have been careful to exclude effects on immunoreactivity when establishing our immunofluorescence readouts. Importantly, while we see that intensity of certain intranuclear stainings decrease, we also observe that the intensities of several intranuclear stainings increase upon HP (Sox9, H3K27me3; Figs 1C and 2G), excluding generalized reduced immunoreactivity after pressure. In addition, key findings (Sox9 increase, gH2AX decrease) were confirmed by immunoblot (Fig. 1B and new Supplementary Fig. 4A). Finally, new qPCR analyses of cell cycle regulators show strong upregulation of P21 (new Fig. 1K), indicative of quiescence and thus further consolidating the EdU incorporation data showing reduced replication.

2. For Fig.1: has Sox9 immunostaining been verified by immunoblot? The increase in Col2A1 mRNA is only ~50%; is that refected in protein change and is it biologically significant? Does nuclear area vary with cell cycle stage (as relevant to Fig.1F,G and 2B,C?

Sox9 was indeed verified by immunoblot (moved to main Fig. 1B). We have further included Col2A1 immunofluorescence analysis, which shows increased protein expression to strengthen this aspect (new Supplementary Fig. 1D). Also, as suggested we have quantified nuclear size in EdU-positive and EdU-negative cells. This analysis shows that EdU-negative cells have smaller nuclei (new Supplementary Fig. 2B), which is consistent with observations that nuclei are bigger in G2/S phase of the cell cycle (Neumann and Nurse, 2007). However, this expected correlation holds true both for HP and control cells. Importantly, HP nuclei are smaller than CTR nuclei independent of EdU incorporation status (new Supplementary Fig. 2B), indicative of a non-cell cycle related mechanism of nuclear size regulation, prompting us to analyze heterochromatin. We thank the reviewer for helping us to clarify this.

3. High hydrostatic pressure in cartilage is balanced by hyperosmotic pressure, which is a better way to say with "high water content, chondrocytes within the cartilage are exposed to high levels of hydrostatic pressure". Importantly, for chondrocytes, hyperosmotic pressure indeed has a similar effect as here "Hydrostatic pressure triggers nuclear shrinkage" as previously quantified for hyperosmotic stress in Irianto (Biophys J 2013) - as should be cited. The same paper also provided EM and additional evidence that 'Osmotic Challenge Drives Rapid and Reversible Chromatin Condensation in Chondrocytes'. Kinetics and reversibility were clarified in that paper; are the changes here similarly reversible and rapid?

We appreciate this expert feedback and have included discussion of this aspect along with the suggested citation on p. 6. Our live imaging of nuclear size shows immediate nuclear size change upon pressurization (Fig. 2B) consistent with the observations by Irianto et al (Irianto et al., 2013). We have further performed additional experimentation to assess reversibility and show that both H3K9me3 heterochromatin, EdU incorporation and replication stress (gH2AX) are restored to steady state within 3h after halting pressurization (new Fig. 2I and 4E).

4. Fig.3 is not especially novel: Peters (Cell 2001) showed in mice that Loss of the Suv39h Histone Methyltransferases Impairs Mammalian Heterochromatin and Genome Stability.

We thank the reviewer for pointing out this relevant publication, which we also had cited in the manuscript. It is important to note that our observations differ from the effects reported in this previous study. Peters et al report that complete loss of Suv39 impairs genome stability at pericentric heterochromatin. In our cells H3K9me3 is decreased but not completely lost. Thus, instead of increased genome instability, we observe reduced DNA damage, which we attribute to decreased replicative stress that we observe in these cells (Fig. 4A-C). Thus, our observations bring novel insights to expand previous work by us and multiple other laboratories (Discher, Lammerding, Piel etc), how mechanical stress impacts genome maintenance through a panel of mechanisms and in a highly cell type specific manner. We show that physiological levels of hydrostatic pressure in

© 2020. Published by The Company of Biologists under the terms of the Creative Commons Attribution License (https://creativecommons.org/licenses/by/4.0/).

chondrocytes reduce DNA damage and this, potentially highly physiologically relevant effect has not been described before.

5. Fig.4A,4B,5B: is gH2AX verified by immunoblot (to avoid pressure artifacts on staining)? Normalize gH2AX by DNA per nucleus to determine whether the DNA damage density is affected by pressure.

We have verified gH2AX by immunoblot (new Supplementary Fig. 4A). As suggested, we have now also normalized gH2AX immunofluorescence intensity with DAPI intensity to show that DNA damage density is indeed decreased by pressure (new Supplementary Fig. 4B).

6. Fig.4I: Does overexpression of Suv39H1 fully rescue High Pressure effects? If partial rescue, then why?

Our careful assessment of the data is that the rescue is complete, as cells with Suv overexpression show no difference in DNA damage with or without HP (Fig. 4J). Suv overexpression itself, in the absence of HP, decreases DNA damage slightly, which might be expected due to the central role of heterochromatin in DNA damage repair and replication (Goodarzi et al., 2008; Liu et al., 2013). We have edited the manuscript to better bring across this point.

7. The authors state that "Lamin A/C levels were not significantly altered in response to HP (Supplementary Fig. 2B)". It should be cited that Lamin A/C has been previously reported to correlate with and respond to tensile stiffness (Swift 2013) rather than bulk compression as applied here.

We thank the reviewer for pointing out this important point. We have now incorporated this clarification along with the citation.

8. I have missed evidence for the statement: "promoting replicative stress by inhibition of topoisomerase II triggers chondrocyte differentiation".

Knockdown of Topo-II would also seem necessary.

This statement was based on the observation that etoposide decreased Sox9 expression. We have rephrased this statement to “promoting replicative stress by inhibition of topoisomerase II decreased Sox9 expression, suggesting that it enhances chondrocyte differentiation" to avoid overinterpretation of this result. We further agree that Topo-II depletion instead of inhibition would be more conclusive but unfortunately complete removal of Topo-II compromises cell viability so we were unable to carry out this experiment.

Minor:
1. Statistical significance needs to be scrutinized for bars with overlapping errors, including Fig.2A,4C,S2E.

We have carefully scrutinized the statistical analyses and they are correct. We have altered the data presentation to scatter plots to better illustrate the differences.

Reviewer 2

Advance Summary and Potential Significance to Field:
This study by the Wickstrom lab utilizes several bespoke platforms for applying differing levels of hydrostatic pressure to chondrocytes. They show that high hydrostatic pressure (HP) promotes chondrocyte quiescence and identity. They show this state is at least associated with chromatin decompaction and increase in H3K27me3-marked facultative heterochromatin, and induced by a reduction in H3K9me3-marked constitutive heterochromatin. They finish by showing that the main mechanism for the maintenance of the chondrocyte progenitor state is reduction of replicative stress, associated with a reduction of H3K9me3.

This is a clear, interesting, and important paper that continues along a research thread, exemplified by the Wickstrom lab, showing a relationship between mechanical stress and

© 2020. Published by The Company of Biologists under the terms of the Creative Commons Attribution License (https://creativecommons.org/licenses/by/4.0/).

chromatin modifications that mitigate DNA damage and affect transcription in progenitor cells. I have a number of comments, but, importantly, they are minor, and aside from potentially a quantification in point 1 and perhaps a small experiment in point 2, I would not expect any of them to require additional experiments.

We thank the reviewer for this positive assessment of our study and are grateful for the expert feedback that helped us to further strengthen our manuscript.

1. I'm a little uncomfortable with the implication that overall higher levels of Sox9+ in a cell would make it a better progenitor cell. At the least, it might be interesting to supplement the data in Fig. 1C with a quantification of + and 1, binning in thirds, for example, and examining how many + and - cells there are (using the bottom and top third, for example). I just don't know what it means to express Sox9 more in a cell that already expresses it, when TFs could potentially just be in excess at some point.

We appreciate this thoughtful feedback. Instead of just showing means of independent experiments we have altered the plots to show full range of the intensity measurements. Inspection of the data distribution shows that there is a range of Sox9 intensities in single cells, instead of separated populations of Sox9-positive and -negative cells (new Fig. 1D and 3E), confirming that Sox9 expression is increased by HP. Importantly, it has been shown that Sox9 transcriptional effects are dose dependent and for example Sox9 overexpression further enhances its biological function, whereas haploinsufficiency leads to compromised function, indicating that cells are sensitive to Sox9 protein level changes (Panda et al., 2001; Lefebvre and Dvir- Ginzberg, 2017)

In further support of the impact of HP on cell identity we have analyzed a broad set of progenitor markers by qPCR to show that they are increased upon HP, suggesting that the increased Sox9 expression reflects the overall differentiation status of the cell which is affected by HP. Also the quiescence gene P21, which is increased upon HP, is in fact a direct target gene of Sox9 (Panda et al., 2001). However, we have been very careful not to imply any causation between Sox9 and the other cell state markers, but interpret them collectively as indication of a progenitor cell state.

2. It should be checked whether apoptosis or live/dead ratio is changed upon HP.

We thank the reviewer for this suggestion and have analyzed apoptosis and necrosis in cells exposed to HP. These new experiments show that HP does not trigger cell death (new Supplementary Fig. 1F, G)

3. I'm a little confused about the role of chromatin decompaction here. Why would this correlate with a loss in nuclear size? Maybe I'm not sufficiently up on the literature, but doesn't it seem it should be the opposite? You're increasing the entropy which should relieve the entropic pressure, at least locally. If you are applying an external pressure that should more than compensate, but the Suv39h1 depletion leading to loss of nuclear size is surprising to me because there's no compensating HP. To me this is a genuinely surprising result worthy of further comment, if possible. Same goes for role of chromatin compaction (via H3K27me) in size increase.

We are grateful for the expert comment and realize that a more detailed discussion of this finding is important. As the reviewer points out, bulk decompaction of chromosomes has been suggested to increase nuclear volume by increasing entropic pressure (Mazumder et al., 2008). While we think that the idea of entropic pressure is intriguing, it is also reasonable to postulate that the volume changes resulting from chromatin decompaction could be buffered by deformation of nucleocytoplasmic components and water exchange through nuclear pores or other more complex mechanisms. The more fine-tuned remodeling of heterochromatin that we observe (decreased H3K9me3 but increased H3K27me3), which also specifically occurs in close proximity of the nuclear lamina in response to HP, could in particular involve such more complex regulation. Importantly, whereas both H3K9me3 and H3K27me3 compact chromatin, only H3K9me3 anchors chromatin to the lamina. This is likely to affect the apparent tension of the nuclear envelope as demonstrated by us recently (Nava et al., 2020). Thus, we propose that HP-induced decrease in H3K9me3 particularly at nuclear periphery affects lamina stiffness and membrane tension to decrease the nuclear volume (Enyedi and Niethammer, 2017). This discussion has been included in the

© 2020. Published by The Company of Biologists under the terms of the Creative Commons Attribution License (https://creativecommons.org/licenses/by/4.0/).

manuscript.

4. It's really not clear that H3K27me3 increases are compensatory. The data on the role of Ezh2/H3K27me3 are not super conclusive and the feedback between these two methylation marks is not understood all that well. I'm not a connoisseur so I could be wrong and I'm happy to be corrected on this if so. Moreover, the specificity of these enzymes, in particular Suv39h1, is pretty high, but it's unclear, at least to me, whether or not other enzymes could play a compensatory role upon depletion of the named ones. Could the authors comment? Words like 'responsible' are used without modifiers so I just want to make sure this is warranted by the data. Also, not sure about statement 'whereas increased H3K27me3 likely represented a compensatory effect with a possible role in nuclear volume regulation' - why would it have a role in nuclear volume regulation? This is similar to point 3. There could be quite a bit going on here.

We see the point of the reviewer. Although we in our previous work, using biaxial stretch on epidermal stem cells demonstrate compensation of H3K9me3 by H3K27me3 on a gene-by-gene level using chromatin immunoprecipitation (Le et al., 2016), we have not carried out such in-depth analysis here and should apply caution in extending the conclusions to this work. We have carefully edited the manuscript to avoid definitive expressions of causality where this has not been comprehensively demonstrated.

5. It might be good to scale back conclusions about replicative stress leading to differentiation. This seems strange (as acknowledged in Discussion) and the data is not highly conclusive without more support. I wouldn't recommend pushing the point further, it's an interesting observation, but the language should reflect that. Simply using words like 'suggest' as opposed to 'show' helps, and more of an acknowledgement in conclusions about potential weaknesses in current data for that.

We agree and have rephrased this statement "promoting replicative stress by inhibition of topoisomerase II decreased Sox9 expression, suggesting that it enhances chondrocyte differentiation" to avoid overstating the conclusion.

6. On that same point, there are a few examples where the authors are verging on overstatement based on the data. It is not blatant, but given that the data are extremely interesting in and of themselves and will represent an important contribution to the literature it would be good to be extra careful about this. An example is the line 'To dissect the possible causative relationship between quiescence, replicative stress and chondrocyte identity, we asked if decreasing replication could reduce replicative stress/DNA damage'. I don't think that serum starvation and Fig. 5D, E are showing anything causative since serum starvation is a blunt tool and Fig. 5D, E are correlative.

We have carefully edited the manuscript to avoid definitive expressions of causality where this has not been comprehensively demonstrated.

7. Not sure what 'Thus, Ca2+ signaling is through mechanosensitive channels is a likely key mediator of the cellular and nuclear effects of a number of extrinsic forces, including stretch, compression, and HP, suggesting existence of a generic mechanoresponse across various extrinsic forces.' means. Could the authors clarify?

We realize that this sentence was unclear. We intended to propose that Ca2+ signaling might be involved in a broad range of mechanoresponses. Also in light of also the additional comment of the other reviewer of applying caution in linking all effects of HP to mechanical stress we have rephrased this statement to read: "Thus, Ca2+ signaling through mechanosensitive channels is likely a key mediator of the cellular and nuclear effects of a number of stimuli that induce mechanical stress and/or osmotic changes, including stretch, compression, and HP".

8. It's at least implied at several points that mechanical stress is causing the changes seen. HP certainly increases mechanical stress, but it could be the volume changes themselves that lead to the changes, as one would see with increasing osmolarity. So in this sense, the effect of mechanical stress is indirect. Granted, it doesn't matter that much in the grand scheme of things, but I think it's important to be precise on this point particularly in the discussion.

© 2020. Published by The Company of Biologists under the terms of the Creative Commons Attribution License (https://creativecommons.org/licenses/by/4.0/).

We agree and have carefully gone through the manuscript to ensure that we do not make a direct link from hydrostatic pressure to mechanical stress.

Reviewer 3

How mechanoregulation of chondrocyte differentiation states and the associated molecular mechanisms impact on chondrocyte fate is an important biological question relevant to cartilage development and skeletal diseases.
Hydrostatic pressure is considered as one of the most important mechanical stimuli for cartilage. In this paper Maki K. et al aim to provide molecular insight into the mechanism how mechanical loading affects chondrocyte differentiation and maintenance. They investigated the impact of hydrostatic pressure (exerted via a custom made pressure chamber) on chromatin modification states of isolated primary chondrocytes and the ATDC5 chondrogenic cell line. Quantitative measurements were made of nuclear changes by live imaging experiments. The role of changes in histone modification on chondrocytes was assessed by investigating the impact of manipulating histone modifications by depleting Suv39H1 H3K9me 3 methyl transferase using siRNA.
The association of histone modification changes with replicative stress and the impact on chondrocyte cell state and differentiation was also studied.
They conclude that mechanical stimuli promotes chondrocyte quiescence and progenitor state via inducing chromatin decompaction and reducing replicative stress. In general the quality of the data is high and the paper well written. However there are a number of major issues which limit the scope of the bold interpretation and conclusions regarding the mechanistic link invoking heterochromatin remodeling, replicative stress and chondrocyte quiescence and progenitor state as stated in the title.

We thank the reviewer for the positive assessment of our manuscript and the constructive expert advice that helped us to further strengthen it. We have addressed the expert criticism of the reviewer by a panel of new experiments as well by carefully editing the text to avoid overstatement.

Major concerns
1. There are several major caveats limiting the interpretation of the data and in vivo relevance of the findings. The first caveat is related to the chondrocyte population studied. Assessments of changes in gene expression were done by qRT-PCR. However this is limited by the fact that it is likely that the starting population of chondrocytes isolated from articular cartilage will be heterogeneous in their differentiation state, there are possibly perichondrium cells present and additionally the cells are transformed by SV40 T antigen which will impact on gene expression and their chondrocyte identity. In addition, cells in the surface and medial zones of articular cartilage may respond differently to the mechanical loading. Sub-fractionation of the starting chondrocytes may be an option, or validating the observed changes in heterochromatin and the expression of progenitor markers in intact articular cartilage. An additional question arising is to what extent does the SV40 T antigen immortalization induce the reactivation of DNA synthesis in the nuclei of non-dividing articular chondrocytes (PMID: 2548682)? This is relevant to the Edu and DNA replication experiments.

We thank the reviewer for these expert comments. Indeed this is a cell biological study, and thus comprehensively addressing the in vivo relevance of our observations remains open for further investigation.

We nevertheless fully agree that the issues of heterogeneity and immortalization are important points and we have addressed these issues with a panel of new experiments and analyses:

1. We have carefully analyzed the starting population of cells and observe that they are all Sox9-positive. We now present the full range of single cell intensity data to demonstrate the absence of a Sox9-negative population (new Fig. 1D and 3E).

2. We have included immunofluorescence analysis of Col2a2, which shows a homogeneous pattern (new Supplementary Fig. 1D).

© 2020. Published by The Company of Biologists under the terms of the Creative Commons Attribution License (https://creativecommons.org/licenses/by/4.0/).

3. To exclude that immortalization of chondrocytes would affect their response to HP we have repeated key aspects of the study with freshly isolated immortalized chrondrocytes. Here, articular cartilage was carefully dissected and single cells were isolated and plated on feeder cells. After removing feeders and confirming that all cells were positive for Sox9, we plated these cells on analysis plates without further passaging. Importantly, these freshly isolated chondrocytes behaved similar to immortalized cells in that Sox9 expression was increased whereas EdU incorporation was decreased upon HP (new Supplementary Fig. 1E).

4. As suggested we have added additional in vivo analyses of intact mouse cartilage to strengthen the in vitro findings. We showed already in the previous manuscript version that close to the articular surface of mouse cartilage, where HP is high, and also high levels of Sox9 are found, cells have low levels of proliferation and DNA damage. In addition, DNA damage and proliferation correlate, as in the in vitro experiments (Fig. 5D, E). We now further show, consistent with the in vitro data, that H3K9me3 levels are low close to the articular surface (new Fig. 5F).

2. Using qRTPCR for Sox9, Acan1 and Col2a1 alone as proof of chondrocyte identity is not sufficient. Validation of qRTPCR results should be done by immunostaining/ in situs on the cells themselves. Additional assessments for genes marking different differentiation states (proliferating, hypertrophic), cell cycle status and testing for the expression not characteristic of chondrocytes such as Col1a1 should be included. The data presented in Supplementary Fig.1 - alcian blue staining lack cellular resolution and hence hetereogeneity in gene expression cannot be assessed. Staining for SOX9 and other markers would be more convincing to show the chondrocyte identity of the cells. In Fig.1 B it appears that there are more SOX9 expressing cells in the HP sample and the intensity in individual cells varies. It would be informative to quantify the number of SOX9 positive cells.

As suggested, we have included immunofluorescence analysis for Sox9 and Col2a1 and show that the isolated chondrocytes show higher protein levels of these key identity markers compared to the widely used chondrocyte-like cell line ATDC (new Supplementary Fig. 1B).

Additionally, as discussed in the previous response, instead of just showing means of independent experiments we now also show the full range of the Sox9 intensity measurements. These show that there is a range of Sox9 intensity in single cells rather than two separate populations of Sox9-positive and -negative cells (new Fig. 1D), and that Sox9 expression is increased by HP, instead of the pool of Sox9- expressing cells being increased.

Finally, we have added qPCR analysis of additional chondrocyte progenitor markers with direct comparison to ATDC5 cells to confirm the identity of these cells (new Supplementary Fig. 1A).

3. SOX9 is also known to promote chondrocyte proliferation, but there seems to be a dichotomy between number of Edu cells and number of SOX9 expressing cells in Fig, 3 E-G. Please explain if transcription is down and SOX9 expression (immunostaining) is upregulated - is this effect at translation/protein stability or transcription. The impact of Suv39H1 knockdown on the number of SOX9 expressing cells and on chondrocyte identity/differentiation status and quiescent state should be shown using molecular markers.

We appreciate the reviewer's expert comments. Although it has been demonstrated that Sox9 is required for chondrocyte proliferation and hypertrophy (Ikegami et al., 2011; Dy et al., 2012), Sox9 has also multiple additional functions in maintaining chondrocyte lineage identity and is also expressed in quiescent chondrocytes in vivo (Lefebvre, 2019). Importantly, overexpression of Sox9 in chondrocytes has been shown to trigger quiescence through P21 expression (Panda et al., 2001). We have added new data to show that P21 is strongly upregulated upon HP (new Fig. 1K), consistent with the notion of quiescence and increased Sox9 expression. Importantly, however, we do not suggest any causality between Sox9 and replication/quiescence, we show that HP both reduces replication as well as increases Sox9 levels, along with increasing the levels of other progenitor markers and P21 (Fig. 1D and new Fig. 1K). Thus, our results are not in contradiction with previous literature.

© 2020. Published by The Company of Biologists under the terms of the Creative Commons Attribution License (https://creativecommons.org/licenses/by/4.0/).

The question of Sox9 expression regulation is interesting, but highly complex. Sox9 is regulated both on transcriptional and posttranslational levels through mechanisms that are only partially understood (Lefebvre and Dvir-Ginzberg, 2017). For example, it has been shown that Sox9 protein is substantially stable and outlives Sox9 mRNA that has an extremely short half life, for example in hypertrophic chondrocytes (Tew and Clegg, 2011). We have carried out analyses of Sox9 mRNA levels in response to HP and indeed do not observe substantial upregulation. This suggests that the regulation of Sox9 by HP is complex and could be posttranscriptional . As there are multiple possible post transcriptional mechanisms by which HP could regulate Sox9, we conclude that although highly interesting, understanding these mechanisms would be a project of its own and therefore beyond the scope of the current manuscript.

As suggested, we have included analysis of chondrocyte progenitor and differentiation markers upon Suv knockdown. These new data show that Suv knockdown results in very similar mRNA changes as HP, namely increased Mcam, unchanged Sox9 and decreased Col1a1 (new Fig. 3F).

4. As in vivo cartilage is exposed to stresses between 0.2 – 10 MPa, do the cells respond differently to different levels of pressure? In this study the hydrostatic pressure in the study is stated as set to 1 MPa. There should be discussion comparing this work with a previous study on ATDC5 where changes in the expression of Polycystins, SOX9, and RUNX2 were found in response to constant hydrostatic pressure of 14.7 kPa (PMID: 27909759).

We thank the reviewer for this suggestion to comment on the previous study on ATDC5 with constant pressure. It is important to note that the ATDC5 cells are a teratocarcinoma cell line, which itself could already explain the different experimental outcomes. Further, constant versus cyclic pressure are two fundamentally different experimental setups, so comparison of these two studies is challenging. We chose to apply cyclic pressure as this is the physiological mode of joint mechanical loading.
We have added a discussion of these differences in the Discussion section of the manuscript.

5. A major omission of the analyses is the inclusion of indicators of established mechanosensitive pathways, such as MRTF/SRF and Hippo signaling

We fully agree that the MRTF and Hippo signaling pathways are central regulators of mechanotransduction. However, it is important to note that they are not the only downstream mediators of mechanical signals, and recent work highlight the role of the nucleus as a mechanosensor independent of these major pathways (Maurer and Lammerding, 2019). We focused our work on the effects of pressure on chromatin, also as we have previously observed effects of mechanical force on global transcription that cannot be explained by the effects of a single transcriptional pathway such as MRTF or YAP (Le et al., 2016). This decision was further guided by experiments that we carried out at the onset of this project where we did not observe any effects of HP on the YAP (Hippo) pathway or the actin cytoskeleton (MRTF).

6. A major focus of the paper is to correlate the changes in chromatin modification induced by HP with transcription, replication and DNA damage and a quiescence state of the chondrocytes. What is the molecular basis of concluding that the chondrocytes are quiescent? Is this because there is nuclear shrinkage? Is it possible that the chondrocytes have entered a senescent state in response to HP? It would be important to define the quiescent versus senescent state by molecular markers (see e.g. PMID: 29044508)

We made the conclusion that hydrostatic pressure promotes quiescence based on reduced replication (EdU incorporation), cell cycle stage (DNA intensity vs EdU analyses) and low transcriptional activity. To further strengthen this conclusion, we have now analyzed mRNA levels of P21, which has been shown to regulate stem cell quiescence (Panda et al., 2001) as well as P27 which is involved both in senescence and quiescence (Zhao et al., 2016; Miller et al., 2007), and P16 which is a senescence marker (Diekman et al., 2018). Interestingly, P21 is strongly upregulated upon HP, whereas P27 and P16 are not strengthening our conclusion that HP triggers quiescence (new Fig. 1K). We thank the reviewer for this helpful suggestion.

7. The logic of concluding that reduced γH2AX in HP was secondary to reduced transcription and replication (page 6) is not clear. Reduced Edu incorporation should reflect reduced replication and

© 2020. Published by The Company of Biologists under the terms of the Creative Commons Attribution License (https://creativecommons.org/licenses/by/4.0/).

therefore less replicative stress?

We apologize for the lack of clarity on this aspect. Indeed, as the reviewer suggests, we propose that reduced EdU incorporation reflects reduced replication and therefore less replicative stress. We have edited the manuscript to make this point clearer.

8. Concerns about data presentation:
Figure 4A the levels of γH2AX are decreased upon HP not increased.

We thank the reviewer for the careful examination of the data. Based on the data in Fig. 4A we indeed conclude that HP decreases *γH2AX*.

In Figure 4B, which is CTR and HP?

Both images were CTR as HP showed little γH2AX. To avoid confusion we have included the corresponding HP panel (new Fig. 4B).

In Figure 4I, the intensity of γH2AX in SUV-GFP with HP condition is higher than SUV-GFP without HP. Does it mean SUV overexpression and HP condition has a negative feedback effect?

Careful quantification of the immunostaining data shows that there is no difference in γH2AX in SUV-GFP cells with and without HP (Fig. 4J), whereas there is a clear difference between GFP and SUV-GFP cells, allowing us to conclude that restoring H3K9me3 prevents the HP from decreasing γH2AX.

In Figure 5D, is it possible to show the chromatin state (H3K9me3, H3K27me3) in in vivo articular cartilage?

We have included quantitative immunofluorescence analyses for H3K9me3 and H3K27me3 in vivo in mouse articular cartilage. We show H3K9me3 intensity is lower at the surface zone compared to the deeper medial zone, supporting our conclusion that H3K9me3 is low in cells experiencing high HP (new Fig 5F). The levels of H3K27me3 were not significantly different at the cartilage surface (new Supplementary Fig. 5A), suggesting that the regulation of this histone mark is more complex and extends beyond the effects of HP.

9. The results obtained in this study should be discussed in the context of the other studies on the hydrostatic pressure in chondrocytes, such as PMID: 28813497, PMID: 27909759, PMID: 28085114, PMID: 26528971. How do the changes in chromatin compare with early studies on chromatin rearrangement during chondrogenesis and C-heterochromatin distribution (PMID: 1927209).

We thank the reviewer for pointing out these interesting studies. We have now added discussion on the differences that can be observed in responses of chondrocytes to the our regime (cyclic, 1MPa in our study) in comparison to the referenced non- physiological hydrostatic pressure (Montagne et al., 2017) as well as physiological pressure (Miyanishi et al., 2006; Li et al., 2016). We hypothesize that the differences in observation arise from these fundamentally different HP regimes as well as the differences in cell types used.

The reference *PMID: 1927209* (Ērenpreisa et al., 1991) analyzes DNA intensity to quantify chromocenters, which are clusters of centromeric and pericentromeric chromatin (shown by others to be enriched with H3K9me3) in chick cartilage. They show that chromocenter intensity increases during differentiation. This is consistent with what we observe in our cell cultures where low H3K9me3 is associated with the progenitor state, and previous large body of data showing that the amount of heterochromatin is low in stem cells and increases upon differentiation.

Minor issues
1. Clarification about quantitation: How the normalization of results shown in the figures was done should be stated. For example how was normalized intensity determined? Examples are the Y axis in the bar charts such as Figure 1C, Figure 2C, 2H etc.

© 2020. Published by The Company of Biologists under the terms of the Creative Commons Attribution License (https://creativecommons.org/licenses/by/4.0/).

Normalization was performed by dividing the measured mean intensities of the HP samples by the mean intensity of the CTR sample. This normalization allowed us to compare intensity measurements from independent experiments, as immunofluorescence has experiment-to-experiment variability. This has been clarified in the Materials and methods section.

2. Inaccurate statements such description of the process of endochondral ossification. There are many publications that show that chondrocytes are now known to survive to become osteoblasts in bone formation (reviewed in PMID: 30949840)

We thank the reviewer for this expert comment, we have amended this statement by removing the reference to apoptosis and including direct transdifferentiating.

3. The sentence on page 6 "immunofluorescence analyses revealed significantly reduced upon yH2AX …" doesn't make sense.

We thank the reviewer for pointing out this typo that we have now corrected by deleting the unnecessary "upon".

4. On page 8 the sentence "As cells are essentially aqueous liquids…." is obviously not accurate.

We have removed this statement.

References

Diekman, B.O., G.A. Sessions, J.A. Collins, A.K. Knecht, S.L. Strum, N.K. Mitin, C.S. Carlson, R.F. Loeser, and N.E. Sharpless. 2018. Expression of p16INK4a is a biomarker of chondrocyte aging but does not cause osteoarthritis. *Aging Cell*. 17:e12771.

Dy, P., W. Wang, P. Bhattaram, Q. Wang, L. Wang, R.T. Ballock, and V. Lefebvre. 2012. Sox9 directs hypertrophic maturation and blocks osteoblast differentiation of growth plate chondrocytes. *Dev. Cell*. 22:597–609.

Enyedi, B., and P. Niethammer. 2017. Nuclear membrane stretch and its role in mechanotransduction. *Nucleus*. 8:156–161.

Ērenpreisa, J., A. Zhukotsky, N. Butusova, J. Ērenpreiss, and T. Arshavskaya. 1991. Accumulation of DNA within chromocentres of terminally differentiating chick embryo chondrocytes. *Acta Histochem*. 90:113–119.

Goodarzi, A.A., A.T. Noon, D. Deckbar, Y. Ziv, Y. Shiloh, M. Löbrich, and P.A. Jeggo. 2008. ATM signaling facilitates repair of DNA double-strand breaks associated with heterochromatin. *Mol. Cell*. 31:167–177.

Ikegami, D., H. Akiyama, A. Suzuki, T. Nakamura, T. Nakano, H. Yoshikawa, and N. Tsumaki. 2011. Sox9 sustains chondrocyte survival and hypertrophy in part through Pik3ca-Akt pathways. *Development*. 138:1507–1519.

Irianto, J., J. Swift, R.P. Martins, G.D. McPhail, M.M. Knight, D.E. Discher, and D.A. Lee. 2013. Osmotic challenge drives rapid and reversible chromatin condensation in chondrocytes. *Biophys. J*. 104:759–769.

Le, H.Q., S. Ghatak, C.-Y.C. Yeung, F. Tellkamp, C. Günschmann, C. Dieterich, A. Yeroslaviz, B. Habermann, A. Pombo, C.M. Niessen, and S.A. Wickström. 2016. Mechanical regulation of transcription controls Polycomb-mediated gene silencing during lineage commitment. *Nat. Cell Biol*. 18:864–875.

Lefebvre, V. 2019. Roles and regulation of SOX transcription factors in skeletogenesis. *Curr. Top. Dev. Biol*. 133:171–193.

Lefebvre, V., and M. Dvir-Ginzberg. 2017. SOX9 and the many facets of its regulation in the chondrocyte lineage. *Connect. Tissue Res*. 58:2–14.

Li, Y., J. Zhou, X. Yang, Y. Jiang, and J. Gui. 2016. Intermittent hydrostatic pressure maintains and enhances the chondrogenic differentiation of cartilage progenitor cells cultivated in alginate beads. *Dev. Growth Differ*. 58:180–193.

Liu, B., Z. Wang, L. Zhang, S. Ghosh, H. Zheng, and Z. Zhou. 2013. Depleting the methyltransferase Suv39h1 improves DNA repair and extends lifespan in a progeria mouse model. *Nat. Commun.*

© 2020. Published by The Company of Biologists under the terms of the Creative Commons Attribution License (https://creativecommons.org/licenses/by/4.0/).

4:1868.
Maurer, M., and J. Lammerding. 2019. The Driving Force: Nuclear Mechanotransduction in Cellular Function, Fate, and Disease. *Annu. Rev. Biomed. Eng.* 21:443–468.
Mazumder, A., T. Roopa, A. Basu, L. Mahadevan, and G. V Shivashankar. 2008. Dynamics of chromatin decondensation reveals the structural integrity of a mechanically prestressed nucleus. *Biophys. J.* 95:3028–3035.
Miller, J.P., N. Yeh, A. Vidal, and A. Koff. 2007. Interweaving the cell cycle machinery with cell differentiation. *Cell Cycle*. 6:2932–2938.
Miyanishi, K., M.C.D. Trindade, D.P. Lindsey, G.S. Beaupré, D.R. Carter, S.B. Goodman, D.J. Schurman, and R.L. Smith. 2006. Dose- and time-dependent effects of cyclic hydrostatic pressure on transforming growth factor-β3-induced chondrogenesis by adult human mesenchymal stem cells in vitro. *Tissue Eng.* 12:2253–2262.
Montagne, K., Y. Onuma, Y. Ito, Y. Aiki, K.S. Furukawa, and T. Ushida. 2017. High hydrostatic pressure induces pro-osteoarthritic changes in cartilage precursor cells: A transcriptome analysis. *PLoS One*. 12:e0183226.
Nava, M.M., Y.A. Miroshnikova, L.C. Biggs, D.B. Whitefield, F. Metge, J. Boucas, H. Vihinen, E. Jokitalo, X. Li, J.M. García Arcos, B. Hoffmann, R. Merkel, C.M. Niessen, K.N. Dahl, and S.A. Wickström. 2020. Heterochromatin-driven nuclear softening protects the genome against mechanical stress-induced damage. *Cell*. 181:800-817.e22.
Neumann, F.R., and P. Nurse. 2007. Nuclear size control in fission yeast . *J. Cell Biol.* 179:593–600.
Panda, D.K., D. Miao, V. Lefebvre, G.N. Hendy, and D. Goltzman. 2001. The transcription factor SOX9 regulates cell cycle and differentiation genes in chondrocytic CFK2 cells. *J. Biol. Chem.* 276:41229–41236.
Tew, S.R., and P.D. Clegg. 2011. Analysis of post transcriptional regulation of SOX9 mRNA during in vitro chondrogenesis. *Tissue Eng. Part A*. 17:1801–1807.
Zhao, G., H. Wang, C. Xu, P. Wang, J. Chen, P. Wang, Z. Sun, Y. Su, Z. Wang, L. Han, and T. Tong. 2016. SIRT6 delays cellular senescence by promoting p27Kip1 ubiquitin-proteasome degradation. *Aging (Albany. NY)*. 8:2308–2323.

Second decision letter

MS ID#: JOCES/2020/247643

MS TITLE: Hydrostatic pressure promotes chondrocyte quiescence and progenitor state through heterochromatin remodeling

AUTHORS: Koichiro Maki, Michele M Nava, Clémentine Villeneuve, Minki Chang, Katsuko S Furukawa, Takashi Ushida, and Sara A Wickström
ARTICLE TYPE: Research Article

We have now reached a decision on the above manuscript.

To see the reviewers' reports and a copy of this decision letter, please go to: https://submit-jcs.biologists.org and click on the 'Manuscripts with Decisions' queue in the Author Area. (Corresponding author only has access to reviews.)

As you will see, two of the reviewers are fully satisfied and the third found the revisions to be largely responsive but has a few remaining issues. Since there has already been one full round of revision that two reviewers found satisfactory, I don't want to push for significant expansions to the scope of the paper. Instead, I think that the concerns of Reviewer 3 can be addressed through careful rewriting of the manuscript to acknowledge remaining limitations, supplemented (at your discretion) with focused experiments if they will deepen your message. I hope that you will be able to carry these out, because I would like to be able to accept your paper.

We are aware that you may be experiencing disruption to the normal running of your lab that makes experimental revisions challenging.If it would be helpful, we encourage you to contact us to

© 2020. Published by The Company of Biologists under the terms of the Creative Commons Attribution License (https://creativecommons.org/licenses/by/4.0/).

discuss your revision in greater detail. Please send us a point-by-point response indicating where you are able to address concerns raised (either experimentally or by changes to the text) and where you will not be able to do so within the normal timeframe of a revision. We will then provide further guidance. Please also note that we are happy to extend revision timeframes as necessary.

Please ensure that you clearly highlight all changes made in the revised manuscript. Please avoid using 'Tracked changes' in Word files as these are lost in PDF conversion.

I should be grateful if you would also provide a point-by-point response detailing how you have dealt with the points raised by the reviewers in the 'Response to Reviewers' box. Please attend to all of the reviewers' comments. If you do not agree with any of their criticisms or suggestions please explain clearly why this is so.

Reviewer 1

Advance summary and potential significance to field

Per previous review.

Comments for the author

The revision is responsive.

Reviewer 2

Advance summary and potential significance to field

This paper shows that hydrostatic pressure influences chromatin remodelling and replicative stress/transcription, thereby influencing identity in progenitor cells.

Comments for the author

I am satisfied that the authors have sufficiently addressed my concerns. I am happy to support this very interesting paper being accepted as is.

Reviewer 3

Advance summary and potential significance to field

How mechanoregulation of chondrocyte differentiation states and the associated molecular mechanisms impact on chondrocyte fate is an important biological question relevant to cartilage development and skeletal diseases.
Hydrostatic pressure is considered as one of the most important mechanical stimuli for cartilage. In this paper Maki K. et al aim to provide molecular insight into the mechanism how mechanical loading affects chondrocyte differentiation and maintenance. They investigated the impact of hydrostatic pressure on chromatin modification states of isolated primary chondrocytes and the ATDC5 chondrogenic cell line. Quantitative measurements were made of nuclear changes by live imaging experiments. The role of changes in histone modification on chondrocytes was assessed by investigating the impact of manipulating histone modifications by depleting Suv39H1 H3K9me 3 methyl transferase using siRNA. The association of histone modification changes with replicative stress and the impact on chondrocyte cell state and differentiation was also studied. They conclude that mechanical stimuli promotes chondrocyte quiescence and progenitor state via inducing chromatin decompaction and reducing replicative stress. In general the quality of the data is high and revisions made in response to reveiwers. However there are still a few issues which limit the scope of the bold interpretation and conclusions regarding the mechanistic link invoking

© 2020. Published by The Company of Biologists under the terms of the Creative Commons Attribution License (https://creativecommons.org/licenses/by/4.0/).

heterochromatin remodeling, replicative stress and chondrocyte quiescence and progenitor state as stated in the title.

Comments for the author

The authors have improved the manuscript in response to the reviewers' comments. However there are still a few issues outstanding.
1. Claims on quiescence. The Reviewer queried the basis for the authors' main claim that HP induces changes in heterochromatin remodeling which regulates chondrocyte quiescence. As raised previously, key to substantiation of this claim is to demonstrate that the chondrocytes are indeed in a state of quiescence. For this the authors would have to demonstrate that the chondrocytes are indeed in quiescence and distinguish whether or not they might have become senescent. Although some attempt is made to address this point raised by the reviewer with new data in Fig 1K, this conclusion is based on the upregulation of P21 and not p27 and P16. However the literature regarding distinguishing between quiescence and senescence is complex and other markers should be used (e.g. PMID: 29044508, e.g. ARID5A; PMID: 30835892, PMID: 28691365).
2. Chondrocyte progenitor markers, page 4 and elsewhere. The authors have added qRTPCR data for expression of mCAM as diagnostic for a progenitor state when Suv is knocked down. mCAM is one of several markers expressed by osteogenic "metaphyseal mesenchymal progenitors" (PMID: 29230039) just below the growth plate and may not be the most appropriate marker for the progenitor state for the articular chondrocytes being studied here. Indeed a single marker may not be sufficient to definitely conclude the identity/state of the chondrocytes and markers for different chondrocyte differentiation states e.g Ihh, PTH1R, Mmp13 and Col10a1 for example, would be important to assess if the chondrocytes were in a progenitor state or just more differentiated. In that regard page 8 "…suggestive of differentiation" and " might trigger chondrocyte differentiation." Are too vague- what differentiation state? Resting maturing, prehypertrophic, hypertrophic? Taking decrease in Sox9 expression alone is not sufficient as SOX9 is expressed in all those chondrocyte populations except hypertrophic ones.
3. The new data on immunofluorescence analyses for H3K9me3 and H3K27me3 show only a marginal difference in H3K9me3 staining intensity between surface and deeper zones in vivo, and are not very convincing – how many samples were analysed and could there be section variability?
4. Changes in SOX9 expression. Serum starvation resulted in increased Sox9 expression which the authors suggest may be linked to reduced replication (page 8). Have the authors considered that the upregulation of SOX9 may be due to the stress response induced by serum starvation (PMID: 30024379)? Further it has been shown that NR2F1 directly regulates SOX9 to induce dormancy by integrating epigenetic control of quiescence (PMID: 25636082). The authors may wish to discuss/investigate expression of NR2F1 and this possibility.
5. The authors may wish to tone down the claims made in the title as not all the claims are strongly substantiated.
Minor issues.
Page 6. New sentence "Interestingly, the HP-induced changes ? nuclear size, …."
Page 10. There is still a problem with the statement that "As cells are mostly liquid"

Second revision

<u>Author response to reviewers' comments</u>

Point-by-point response

Reviewer 3

The authors have improved the manuscript in response to the reviewers' comments. However there are still a few issues outstanding.

© 2020. Published by The Company of Biologists under the terms of the Creative Commons Attribution License (https://creativecommons.org/licenses/by/4.0/).

We are happy to see that the reviewer was overall satisfied with our extensive revisions. We have addressed the few remaining issues with additional experiments and changes in the text as outlined in detail below.

1. Claims on quiescence. The Reviewer queried the basis for the authors' main claim that HP induces changes in heterochromatin remodeling which regulates chondrocyte quiescence. As raised previously, key to substantiation of this claim is to demonstrate that the chondrocytes are indeed in a state of quiescence. For this the authors would have to demonstrate that the chondrocytes are indeed in quiescence and distinguish whether or not they might have become senescent. Although some attempt is made to address this point raised by the reviewer with new data in Fig 1K, this conclusion is based on the upregulation of P21 and not p27 and P16. However the literature regarding distinguishing between quiescence and senescence is complex and other markers should be used (e.g. PMID: 29044508, e.g. ARID5A; PMID: 30835892, PMID: 28691365).

We agree that it is important to distinguish between quiescence and senescence and further agree that relying on one particular marker is not sufficient. Thus, we have based our conclusion on quiescence on multiple pieces of data:
1. The core definition of senescence is that it is an irreversible cell cycle arrest whereas quiescence is a reversible cell cycle exit (van Velthoven and Rando, 2019). We demonstrate that the changes in replication triggered by HP are reversible (Fig. 2I and 4E).
2. Another hallmark of senescence are senescence associated heterochromatin foci, high in H3K9me3 (Gorgoulis et al., 2019). In contrast to this, histone methylation is generally low in quiescent cells (van Velthoven and Rando, 2019), and we show that H3K9me3 heterochromatin is reduced (Fig. 2D, E).
3. A hallmark of quiescence on the other hand is low transcription and RNA abundance (van Velthoven and Rando, 2019), and we observe low transcriptional activity (Fig 1I, J).

These critical aspects, together with the finding that two hallmark senescence associated genes, p16 and p27, are not upregulated, whereas p21, which is directly linked to chondrocyte quiescence (Panda et al., 2001) is upregulated upon HP, make us confident to conclude that the cells are not undergoing senescence and are instead in a more quiescent state. However, in response to the reviewer's criticism, we have edited the title to avoid overstating the effect of HP on quiescence.

2. Chondrocyte progenitor markers, page 4 and elsewhere. The authors have added qRTPCR data for expression of mCAM as diagnostic for a progenitor state when Suv is knocked down. mCAM is one of several markers expressed by osteogenic "metaphyseal mesenchymal progenitors" (PMID: 29230039) just below the growth plate and may not be the most appropriate marker for the progenitor state for the articular chondrocytes being studied here. Indeed a single marker may not be sufficient to definitely conclude the identity/state of the chondrocytes and markers for different chondrocyte differentiation states e.g Ihh, PTH1R, Mmp13 and Col10a1 for example, would be important to assess if the chondrocytes were in a progenitor state or just more differentiated. In that regard page 8 "…suggestive of differentiation" and " might trigger chondrocyte differentiation." Are too vague- what differentiation state? Resting, maturing, prehypertrophic, hypertrophic? Taking decrease in Sox9 expression alone is not sufficient as SOX9 is expressed in all those chondrocyte populations except hypertrophic ones.

It is important to note that we were not relying on a single marker, as in addition to Mcam we also analyzed markers for the pre-hypertrophic differentiation state (Runx2) and osteogenic differentiation state (Col1A1) which were both downregulated, as would be predicted for a progenitor state. However, we do see the point of the reviewer to be more specific about the differentiation trajectory of the cells and have now included Acan as another marker for immature chondrocytes as well as Pth1r as a marker for pre-hypertrophic chondrocytes. Consistent with our previous data we see Acan increased and Pth1r decreased upon HP. These effects are, albeit less strongly, observed also upon SUV depletion. Thus to confirm the role of SUV39h1 activity and H3K9me3 in attenuating maturation we additionally analyzed the pre-hypertrophic marker Mmp13 in SUV-depleted cells, where it was found significantly decreased, consistent with the other observations. In addition to these new data, and to not overstate the relatively minor transcriptional effects of SUV KD on the progenitor genes, we have edited the text to be more

© 2020. Published by The Company of Biologists under the terms of the Creative Commons Attribution License (https://creativecommons.org/licenses/by/4.0/).

precise about the state of these cells and instead of HP promoting a progenitor state we describe it as preventing or attenuating chondrocyte maturation into the pre-hypertrophic state.

3. The new data on immunofluorescence analyses for H3K9me3 and H3K27me3 show only a marginal difference in H3K9me3 staining intensity between surface and deeper zones in vivo, and are not very convincing – how many samples were analysed and could there be section variability?

We have analyzed multiple sections per mouse from a panel of independent animals (5 mice for H3K9me3 in Fig. 5F and 4 mice for H3K27me3 in Supp. Fig. 5), as also now indicated in Figure Legends, so our dataset is quite extensive. As we blot both the full range and means of independent animals, one can see that the variation is between the animals, indicative of a biological source of variation.
As we describe in the manuscript, HP is only one factor regulating heterochromatin in vivo, and thus it is expected that the differences in histone modifications reflect a number of cellular and molecular events regulating gene expression and chromatin architecture, thus most likely explaining the relatively modest effects.

4. Changes in SOX9 expression. Serum starvation resulted in increased Sox9 expression which the authors suggest may be linked to reduced replication (page 8). Have the authors considered that the upregulation of SOX9 may be due to the stress response induced by serum starvation (PMID: 30024379)? Further it has been shown that NR2F1 directly regulates SOX9 to induce dormancy by integrating epigenetic control of quiescence (PMID: 25636082). The authors may wish to discuss/investigate expression of NR2F1 and this possibility.

We thank the reviewer for this interesting idea. Indeed, mechanical stress commonly activates stress pathways such as p38, so indeed it is possible that both mechanical stress triggered by HP or by serum starvation would involve stress signaling to regulate Sox9 (PMID: 30024379) and that this might occur through NR2F1. The reduction in heterochromatin induced by NR2F1 in cancer cells is indeed intriguing (PMID: 25636082). However, whereas we observe reduced H3K9me3 and increased H3K27me3 upon HP, this study reports a global reduction in both histone marks. Thus, it is clear that the regulation of heterochromatin by stress signaling is complex and most likely cell type-specific, opening an interesting avenue for future research.

5. The authors may wish to tone down the claims made in the title as not all the claims are strongly substantiated.

We have altered the title to “Hydrostatic pressure prevents chondrocyte differentiation through heterochromatin remodeling” to avoid overstatement regarding quiescence or the progenitor state.

Minor issues.
Page 6. New sentence “Interestingly, the HP-induced changes ? nuclear size, ….”

We thank the reviewer for pointing out this typo that we have now corrected by inserting “in”.

Page 10. There is still a problem with the statement that “As cells are mostly liquid”

We are a bit at loss here in terms of what is the problem with this statement. We intend to convey that cells and tissues are considered virtually incompressible due to their high liquid content. We have now changed the statement into “As cartilage tissue and chondrocytes are reported to be incompressible at low strain regime (Bachrach et al., 1998; Ofek et al., 2009), a pressure-sensing mechanism in the absence of a gas-liquid interface remains unclear.”

References
Bachrach, N.M., V.C. Mow, and F. Guilak. 1998. Incompressibility of the solid matrix of articular cartilage under high hydrostatic pressures. J. Biomech. 31:445-451.
Gorgoulis, V., P.D. Adams, A. Alimonti, D.C. Bennett, O. Bischof, C. Bishop, J. Campisi, M. Collado, K. Evangelou, G. Ferbeyre, J. Gil, E. Hara, V. Krizhanovsky, D. Jurk, A.B. Maier, M. Narita, L. Niedernhofer, J.F. Passos, P.D. Robbins, C.A. Schmitt, J. Sedivy, K. Vougas, T. von Zglinicki, D.

© 2020. Published by The Company of Biologists under the terms of the Creative Commons Attribution License (https://creativecommons.org/licenses/by/4.0/).

Zhou, M. Serrano, and M. Demaria. 2019. Cellular senescence: defining a path forward. Cell. 179:813–827.
Ofek, G., D.C. Wiltz, and K.A. Athanasiou. 2009. Contribution of the cytoskeleton to the compressive properties and recovery behavior of single cells. Biophys. J. 97:1873–1882.
Panda, D.K., D. Miao, V. Lefebvre, G.N. Hendy, and D. Goltzman. 2001. The transcription factor SOX9 regulates cell cycle and differentiation genes in chondrocytic CFK2 cells. J. Biol. Chem. . 276:41229–41236.
van Velthoven, C.T.J., and T.A. Rando. 2019. Stem cell quiescence: dynamism, restraint, and cellular idling. Cell Stem Cell. 24:213–225.

Third decision letter

MS ID#: JOCES/2020/247643

MS TITLE: Hydrostatic pressure prevents chondrocyte differentiation through heterochromatin remodeling

AUTHORS: Koichiro Maki, Michele M Nava, Clémentine Villeneuve, Minki Chang, Katsuko S Furukawa, Takashi Ushida, and Sara A Wickström
ARTICLE TYPE: Research Article

I am happy to tell you that your manuscript has been accepted for publication in Journal of Cell Science, pending standard ethics checks.

Reviewer 3

Advance summary and potential significance to field

How mechanoregulation of chondrocyte differentiation states and the associated molecular mechanisms impact on chondrocyte fate is an important biological question relevant to cartilage development and skeletal diseases.
Hydrostatic pressure is considered as one of the most important mechanical stimuli for cartilage. In this paper Maki K. et al provide molecular insight into the mechanism how mechanical loading affects chondrocyte differentiation and maintenance. They conclude that mechanical stimuli antagonizes the differentiation progression and maturation of chondrocytes via inducing chromatin decompaction and reducing replicative stress with implications for the quiescent state.

Comments for the author

The authors have toned down their claims, revised the title and satisfactorily addressed the concerns raised.

© 2020. Published by The Company of Biologists under the terms of the Creative Commons Attribution License (https://creativecommons.org/licenses/by/4.0/).
